# Dietary enrichment of resistant starches or fibers differentially alter the feline fecal microbiome and metabolite profile

**DOI:** 10.1186/s42523-022-00213-9

**Published:** 2022-12-05

**Authors:** Anne H. Lee, Aashish R. Jha, Sungho Do, Elisa Scarsella, Justin Shmalberg, Amy Schauwecker, Andrew J. Steelman, Ryan W. Honaker, Kelly S. Swanson

**Affiliations:** 1 Department of Animal Sciences, University of Illinois at Urbana-Champaign, Urbana, IL 61801 USA; 2 Genetic Heritage Group, Program in Biology, New York University Abu Dhabi, Abu Dhabi, UAE; 3 NomNomNow, Inc., Oakland, CA 94607 USA; 4 PetSmart Proprietary Brand Product Development, Phoenix, AZ 85080 USA; 5 Division of Nutritional Sciences, University of Illinois at Urbana-Champaign, Urbana, IL 61801 USA; 6 Department of Comparative, Diagnostic and Population Medicine, College of Veterinary Medicine, University of Florida, Gainesville, FL 32608 USA; 7 162 Animal Sciences Laboratory, 1207 West Gregory Drive, M/C 630, Urbana, IL 61801 USA

**Keywords:** Feline gastrointestinal health, Feline microbiome, Feline nutrition, Pet health

## Abstract

**Background:**

Cats are strict carnivores but possess a complex gastrointestinal (GI) microbial community that actively ferments dietary substrates that are not digested and reach the colon. The GI microbiota responses to dietary inclusion of resistant starches versus fibers have not been tested in cats. Thus, our objective was to evaluate the effects of diets enriched in resistant starch or fibers on the fecal characteristics, microbiome, and metabolite profiles of cats. Twelve healthy adult domestic shorthair cats (age = 9.6 ± 4.0 year; body weight = 3.9 ± 1.0 kg) were used in a replicated 3 × 3 Latin square design to test diets that were enriched with: (1) resistant starch (ERS), (2) a fiber-prebiotic-probiotic blend (FPPB), or (3) a fiber-prebiotic-probiotic blend + immune-modulating ingredients (iFPPB). In each 28-day period, 22 days of diet adaptation was followed by fecal and blood sample collection. Fecal samples were used for shotgun metagenomic sequencing. In addition, fecal and blood metabolite measurements and white blood cell stimulation was performed to assess immune function.

**Results:**

A total of 1690 bacterial species were identified, with 259 species differing between fiber-rich and ERS treatments. In comparison with fiber-rich treatments that increased diversity and promoted Firmicutes and Bacteroidetes populations, resistant starch reduced microbial diversity and fecal pH, led to a bloom in Actinobacteria, and modified Kyoto Encyclopedia of Genes and Genomes orthology (KO) terms pertaining to starch and sucrose metabolism, fatty acid biosynthesis and metabolism, epithelial cell signaling, among others. Resistant starch also differentially modified fecal metabolite concentrations with relevance to GI and overall host health (increased butyrate; decreased propionate and protein catabolites - branched-chain fatty acids; phenols and indoles; ammonia) and reduced blood cholesterol, which correlated strongly with microbial taxa and KO terms, and allowed for a high predictive efficiency of diet groups by random forest analysis.

**Conclusion:**

Even though domestic cats and other carnivores evolved by eating low-carbohydrate diets rich in protein and fat, our results demonstrate that the feline microbiome and metabolite profiles are highly responsive to dietary change and in directions that are predictable.

**Supplementary Information:**

The online version contains supplementary material available at 10.1186/s42523-022-00213-9.

## Background

Domestic cats are carnivores and have traditionally relied on high-protein, high-fat diets containing relatively low fiber concentrations. Despite having a relatively simple gastrointestinal (GI) tract evolved to digest such diets, they possess a rich GI microbial community that actively ferments dietary substrates escaping host digestion. Several hundred bacterial species, predominated by members of the Firmicutes (36–50%), Bacteroidetes (24–36%) and Proteobacteria (11–12%) phyla, are known to inhabit the feline GI tract [1, 2]. The presence of the above-mentioned phyla and their functions are known to differ among individuals depending on living environment, dietary habits, and other environmental exposures [3], but more research is needed to test how specific factors impact inter-individual GI microbiomes and how they influence host physiology.

Fermentable dietary fibers, carbohydrate-based prebiotics, and resistant starches (RS) are known to influence host GI microbiota and immune function by promoting carbohydrate fermentation, which leads to increases in short-chain fatty acid (SCFA) production. SCFA have many beneficial effects, including improved gut barrier and immune function [4–10]. Other dietary components such as yeast fermentation products and spray-dried plasma (SDP) may serve as immune-modulators using mechanisms unrelated to SCFA production [11–17]. These dietary components are included in pet foods, but with little data on their effects in cats.

Moreover, most of the current microbiota data in cats was obtained from 16 S rRNA gene-based microbial profiling methods. Although those methods provide useful information regarding the microbial populations, they are unable to provide deeper resolution (species or strain) into the microbial community. Furthermore, there is potential for bias during the amplification step for 16 S rRNA methods [18]. Therefore, shotgun metagenomic sequencing has been used to improve resolution and accuracy in recent years and more human studies have applied these methods to gain understanding of diet-induced changes to the microbiota at the taxonomic level as well as the functional features coming from the gene content data [19]. Despite the progress being made in the microbiome field, data in cats are still very limited, with many of the microbiome studies being conducted decades ago, and the impact of diverse diets fed to cats on GI microbial diversity and richness, gene content, and metabolic activity have not been reported. Exploring the microbiota beyond the taxonomic level and evaluating the effects of diet on gene content and its relation to physiological outcomes improve understanding of the mechanistic insights of how microbes potentially affect feline health.

The primary objective of this study was to explore the fecal microbial community profiles and their functions in domestic cats fed diets enriched in either RS or a combination of dietary fibers, prebiotics, and probiotics utilizing shotgun metagenomic sequencing. Secondary objectives were to identify significant fecal bacterial taxa-bacterial gene-metabolite changes in response to the different dietary treatments.

## Methods

### Animals, diets and experimental timeline

The animal study was conducted at Kennelwood, Inc. (Champaign, IL, USA). All animal procedures for this study were approved by the Kennelwood, Inc. Institutional Animal Care and Use Committee (IACUC) and were performed in accordance with the U. S. Public Health Service Policy on Humane Care and Use of Laboratory Animals. Twelve healthy adult female domestic shorthair cats (age = 9.6 ± 4.0 year; body weight = 3.9 ± 1.0 kg) were used in a replicated 3 × 3 Latin square design. All cats were housed in individual pens (54.6 cm W × 64.8 cm L × 68.6 cm H) in an environmentally controlled animal facility. Cats had access to fresh water at all times. Cats were fed once a day to maintain body weight throughout the study. Previous feeding records were used to estimate initial food intake, with weekly body weight and BCS (9-point scale) (both recorded prior feeding) being used to adjust weekly intakes [20].

Three different extruded, experimental kibble diets were formulated to meet all Association of American Feed Control Officials (AAFCO) nutrient recommendations for adult cats at maintenance (Additional file 1: Table S1) [21]. All diets contained a fish-based protein source, grains, and chicken fat, but were formulated to contain unique gut microbiota modulators. The first diet was enriched in resistant starch (ERS) and contained potato flour, a starch source that is more resistant to gelatinization during the extrusion process than grains, providing a source of RS. The second diet [fiber-prebiotic-probiotic blend-containing formula (FPPB)] was formulated to contain a prebiotic (i.e., inulin), a probiotic (i.e., *Lactobacillus acidophilus* and *Enterococcus faecium*), and natural fiber-rich ingredients such as oat groats, beet pulp, and pea fiber. The third diet [fiber-prebiotic-probiotic blend + immuno-modulating ingredient-containing formula (iFPPB)] was formulated similar to FPPB, but with the addition of immunomodulators [i.e., yeast fermentation product (TruMune; Diamond V, Cedar Rapids, IA) and SDP (APC Inc., Ankeny, IA)].

Prior to the study, a veterinary exam was performed and blood samples were collected for serum chemistry and hematology measures to confirm health (see methods below). Each experimental period was 28 d in length, consisting of an adaptation phase from d 1–22, fecal collection phase (e.g., microbiota; metabolites; IgA; pH) from d 23–27, and blood collection on d 28 (e.g., serum chemistry; hematology; immune cell functionality).

### Blood collection and analyses

On d 28 of each experimental period, up to 15 mL of blood was collected via jugular, cephalic, or medial saphenous vein venipuncture for serum chemistry, hematology, and immune assays. Prior to collection, cats were sedated with an intramuscular (IM) injection of a combination of butorphanol tartrate (0.024 mg/kg IM; Torbugesic, Zoetis Inc., USA), dexmedetomidine (0.02 mg/kg IM; Dexdormitor, Zoetis Inc., USA), and ketamine (0.061 mg/kg IM; Zoetis Inc., USA). After blood collection, an injection of the reversal agent for dexmedetomadine, atipamezole hydrochloride (0.2 mg/kg IM; Antisedan, Zoetis Inc., USA), was given. Collected blood samples were immediately placed into appropriate vacutainer tubes: 10 mL in #366,480 BD Vacutainer^®^ glass plasma tubes (Becton Dickinson, USA) for immunoassays, 0.5 mL in #365,974 BD Microtainer^®^ Plastic whole blood tubes with K2EDTA additive (Becton Dickinson, USA) for hematology, and 4.5 mL in #367,974 BD Vacutainer^®^ Plus plastic serum tube with clot activator and gel for serum separation (Becton Dickinson) for serum chemistry. Serum was isolated by centrifugation at 1300 × g at 4 °C for 10 min (Beckman CS-6R centrifuge; Beckman Coulter Inc., USA). Serum chemistry profile and hematology were analyzed using a Hitachi 911 clinical chemistry analyzer (Roche Diagnostics, USA) at the University of Illinois Veterinary Medicine Diagnostics Laboratory.

Immune cell assays were performed as described by Lin et al. [16]. Briefly, peripheral blood mononuclear cells (PBMC) were separated by layering 10 mL of collected blood over Ficoll Histopaque (Sigma, USA) in a 1:1 volume ratio and centrifuged at 300 × g at 4 °C for 30 min. Once PBMC were isolated, the responsiveness of lymphocytes to toll-like receptor (TLR) agonists, including zymosan (TLR2 agonist; 100 µg/mL zymosan; InvivoGen, USA), polyinosinic–polycytidylic acid sodium salt (TLR3 agonist; 50 µg/mL polyinosinic–polycytidylic acid sodium salt, poly(I:C); Sigma, USA), lipopolysaccharides (TLR4 agonist; 100 ng/mL LPS; Sigma, USA) and resiquimod (TLR7/8 agonist; 5 µg/mL resiquimod, InvivoGen, USA) were assessed by measuring tumor necrosis factor-alpha (TNF-alpha) production. Collected PBMC (1 × 10^6^ cells/tube) were stimulated in triplicate in a 96-well plate and incubated for 24 h at 37 °C in 5% CO_2_. Following incubation, collected supernatants were stored at − 80 °C until measurement of TNF-α using a commercial enzyme-linked immunosorbent assay (ELISA) kit (MyBioSource, San Diego, CA, USA).

### Fecal sample collection and metabolite analyses

During the collection phase, total fecal output was collected, and fecal scores were noted. Total feces excreted during the collection phase were taken from the litter boxes, weighed, and frozen at − 20 °C until analysis. All fecal samples during the collection period were subjected to a consistency score according to the following scale: 1 = hard, dry pellets, small hard mass; 2 = hard, formed, dry stool; remains firm and soft; 3 = soft, formed, and moist stool, retains shape; 4 = soft, unformed stool, assumes shape of container; and 5 = watery, liquid that can be poured.

During the fecal collection phase, a fresh fecal sample (within 15 min of defecation) was collected for fecal pH, microbiota and metabolite measurement. Fecal pH was measured immediately using an AP10 pH meter (Denver Instrument, USA) equipped with a Beckman Electrode (Beckman Instruments Inc., USA), and then aliquots were collected. Aliquots for phenols and indoles analysis were frozen at − 20 °C immediately after collection. One aliquot was collected and placed in approximately 2 mL of 2 N hydrochloric acid for ammonia, SCFA, and branched-chain fatty acid (BCFA) analyses. An aliquot of fresh feces was immediately transferred to sterile cryogenic vials (Nalgene, USA), frozen in dry ice, and stored at − 80 °C for microbial analysis. Additional aliquots were used for fresh fecal dry matter (DM) determination and immunoglobulin A (IgA) concentration measurement.

Fecal SCFA (acetate, propionate and butyrate) and BCFA (valerate, isovalerate, isobutyrate) concentrations were determined according to Erwin et al. [22] using a gas chromatograph (Hewlett-Packard 5890 A series II, USA) and a glass column (180 cm × 4 mm i.d.) packed with 10% SP-1200/1% H3PO4 on 80/100 + mesh Chromosorb WAW (Supelco Inc., USA). Nitrogen was the carrier with a flow rate of 75 mL/min. Oven, detector, and injector temperatures were 125, 175, and 180 °C, respectively. Fecal concentrations of phenols and indoles were evaluated using gas chromatography according to Flickinger et al. [23] and fecal ammonia concentration was measured according to the method of Chaney and Marbach [24].

Fecal protein was extracted according to Vilson et al. [25]. Fecal samples (500 mg) were vortexed with 1.5 mL of extraction buffer containing 50 mM-EDTA (ThermoFisher, USA) and 100 µg/L soybean trypsin inhibitor (Sigma, USA) in PBS/L percent bovine serum albumin (Tocris Bioscience, UK). Phenylmethanesulphonyl fluoride (12.5 µL, 350 mg/L; Sigma, USA) was added into each tube and centrifuged for 10 min. The supernatant was collected for measurement of fecal IgA using a commercial ELISA kit (E-20 A; Immunology Consultants Laboratory, USA).

### Diet and fecal chemical composition and digestibility analyses

Fecal samples used for digestibility analysis were dried at 55 °C in a forced-air oven. Diet subsamples and dried fecal samples were ground through a Wiley mill (model 4, Thomas Scientific, USA) through a 2-mm screen. DM and organic matter (OM) content were measured according to the Association of Official Analytical Chemists (AOAC; method 934.01 for DM; method 942.05 for OM) [26]. Crude protein content was calculated from total nitrogen values measured by LECO (TruMac N, Leco Corp., USA; AOAC method 922.15) [26]. Acid-hydrolyzed fat content was determined using methods according to American Association of Cereal Chemists (AACC; method 30−14) [27] and Budde [28]. Total dietary fiber (TDF) was determined for diet and fecal samples according to Prosky et al. [29] and AOAC (method 985.29). Gross energy was measured using a bomb calorimeter (Model 6200, Parr Instruments, USA). Apparent total tract macronutrient digestibility of nutrients and energy were calculated using the following equation:

$$\%\,Digestibility = \frac{{[Nutrient\;intake(g/d) - Fecal\;output(g/d)]}}{{Nutrient\;intake(g/d)}} \times 100\%$$

### Fecal DNA extraction, shotgun metagenomic sequencing, and data analyses

Total DNA was extracted from fecal samples with Zymogen Quick-DNA Fecal/Soil Microbe 96 Mag Bead kit (Zymo Research Corp., USA) using Powerbead Pro (Qiagen, USA) plates with 0.5 mm and 0.1 mm ceramic beads. Extraction controls included water and a characterized homogenized stool. The homogenous stool samples were derived from a mixture of human stool and were used as controls for DNA extraction and library preparation. All samples were quantified with Quant-iT Picogreen dsDNA Assay (Invitrogen, USA). Libraries were prepared with a procedure adapted from the Nextera XT DNA Library Preparation Kit (Illumina, USA). For BoosterShot (Shallow Sequencing, 2 M reads/sample), libraries were sequenced on an Illumina NovaSeq 6000 using single-end 1 × 100 reads (Illumina, San Diego, CA, USA). Library controls included water and DNA from a characterized homogenized stool. Single end shotgun reads were trimmed and processed using a quality control pipeline called Shi7 (version 0.9.9) [30]. The sequences were then aligned to the NCBI RefSeq representative prokaryotic genome collection at 97% identity with BURST using default settings [31]. BURST is a high-throughput DNA short-read aligner that uses several new synergistic optimizations to enable provably optimal alignment in next-generation sequencing datasets. Taxa present in < 5% of samples were removed. The resulting taxonomy table was aggregated at higher taxonomy levels.

Kyoto Encyclopedia of Genes and Genomes (KEGG) orthology (KO) groups were observed directly using alignment at 97% identity against a gene database derived from the strain database used above. KO present in < 5% of samples were removed as part of the quality filtering process. Species richness and Shannon’s diversity index were computed by rarefying samples to various depths starting from 25,000 to 950,000 sequences per sample and increasing sequence depth by 25,000 reads. One hundred iterations were performed at each depth and the mean values were used as the estimate of these measures in each sample. To investigate the effect of treatment on alpha-diversity, the species richness and Shannon’s diversity index were calculated using a rarefaction depth of 950,000. Wilcoxon signed rank test was used to compare the alpha diversity metrics among treatments.

The non-rarefied count data were log-transformed and principal coordinate analysis (PCoA) was performed in R (version 4.0.2) using the Bray-Curtis and Jensen-Shannon distances calculated with the vegan package at the species level [38]. Permutational multivariate analysis of variance (PERMANOVA) was performed using Bray-Curtis distance with 10,000 permutations to assess the differences in community composition using the vegan package [32]. Differential abundance of bacterial phyla, species, and KO terms between treatments was assessed using a negative binomial generalized linear model (GLM) using the differential expression analysis for sequence count data version 2 (DESeq2) package [33]. Taxa with absolute log2 (fold change [FC]) > 2 and adjusted P < 0.01 were considered significant. The adjustment for multiple comparisons was performed using the Benjamini Hochberg false discovery rate (FDR).

Partitioning Around Medoids (PAM) Clustering was performed using the *cluster* package [34]. Individuals were clustered into multiple clusters (K = 1–5) based on the top two PCoA dimensions obtained using Bray-Curtis distances. Goodness of clustering was assessed using a “gap” statistic with 1000 bootstrapped replicates. Random forest classifiers [35] were constructed using the repeated k-fold cross validation and random search implemented in R-package *caret* [36]. The model was trained by optimizing the tuning parameters using a 5-fold cross validation repeated 3 times using species as the predictor and accuracy was used to select the optimal model. The performance of the classifiers was assessed by generating area under the receiver operating characteristic curves (AUC) using the R-package *ROCR* [37].

### Statistical analyses

Except microbiota analyses, all data were analyzed using the Mixed Models procedure of SAS (version 9.4; SAS Institute, Inc., Cary, NC, USA), with treatment considered as a fixed effect and cat and period considered random effects. Data were tested for normality using the UNIVARIATE procedure of SAS. Differences between treatments were determined using a Fisher-protected least significant difference with a Tukey adjustment to control for experiment-wise error. A probability of *P* < 0.05 was accepted as statistically significant with *P* < 0.10 considered trends. Reported pooled standard errors of the mean (SEM) were determined according to the Mixed Models procedure of SAS. Correlation analyses between fecal microbiota and metabolites were assessed by Spearman’s rank correlation test in RStudio (version 1.1.463). Significance was set at FDR adjusted *P* ≤ 0.05.

## Results

### Food intake, body weight, body condition score, and apparent total tract energy and macronutrient digestibility

During this study, cats were fed to maintain their body weight and body condition score. Average daily food intake across all groups was 58.3 g/d, with food intake being lower (*P* < 0.05) in cats fed ERS than those fed iFPPB, but not different from FPPB (Additional file 1: Table S2**)**. While body weights were not drastically altered, a greater level of food refusals occurred in cats fed ERS. Caloric intake was lower (*P* < 0.05) in cats fed ERS than those fed FPPB or iFPPB and lower (*P* < 0.05) in cats fed FPPB than those fed iFPPB. Cats fed ERS had slightly lower (*P* < 0.05) body weight compared to those fed FPPB, but not different from iFPPB. Body condition score (BCS) did not differ among dietary treatments. Apparent total tract digestibility of DM, OM, crude protein, fat, and energy were lower (*P* < 0.05) in cats fed ERS than those fed FPPB or iFPPB (Table 1). Total dietary fiber digestibility was higher (*P* < 0.05) in cats fed iFPPB than those fed ERS or FPPB. Overall, there were statistical decreases in food intake, body weight, and macronutrient digestibility in cats fed ERS compared with other groups. However, the decrease in body weight had a minimal effect on BCS and no negative health outcomes were observed throughout the study.

**Table 1:** Feline fecal characteristics and metabolite concentrations and apparent total tract nutrient digestibility of experimental diets

	Dietary treatment			SEM	*P*-value	
	ERS^1^	FPPB	iFPPB		Treatment	ERS vs. FPPB and iFPPB
*Characteristics *						
pH	4.87^a^	5.63^b^	5.97^b^	0.12	< 0.0001	< 0.0001
Fecal score^2^	3.6^b^	3.2^ab^	2.7^a^	0.20	0.0001	0.0002
Fecal DM (%)	25.6^a^	28.2^ab^	31.6^b^	1.26	0.0008	0.0013
*Digestibility *	%					
Dry matter	72.0^a^	78.4^b^	76.9^b^	1.380	< 0.01	< 0.01
Organic matter	73.8^a^	82.2^b^	81.4^b^	1.305	< 0.0001	< 0.0001
Crude protein	68.0^a^	80.3^b^	83.3^b^	1.295	< 0.0001	< 0.0001
Acid-hydrolyzed fat	87.7^a^	90.0^b^	91.4^b^	1.076	< 0.01	< 0.01
Total dietary fiber	30.4^a^	30.0^a^	39.3^b^	2.918	0.05	0.25
Energy	74.3^a^	83.1^b^	83.1^b^	1.270	< 0.0001	< 0.0001
*Metabolites *	µmol/g DM					
Total SCFA	554.1	602.6	529.2	35.66	0.3583	0.7859
Acetate	290.0	353.0	329.1	23.36	0.1101	0.0498
Propionate	51.9^a^	125.1^b^	139.8^b^	6.69	< 0.0001	< 0.0001
Butyrate	212.2^c^	124.5^b^	60.3^a^	18.40	< 0.0001	< 0.0001
Total BCFA	36.3^a^	50.5^b^	41.0^ab^	3.77	0.0417	0.0478
Isobutyrate	1.9^a^	4.5^b^	5.8^b^	0.49	< 0.0001	< 0.0001
Isovalerate	3.7^a^	6.6^b^	8.5^b^	0.70	0.0002	0.0001
Valerate	30.7^ab^	39.5^b^	26.8^a^	3.36	0.0370	0.5508
Total phenols and indoles	0.05^a^	1.18^b^	1.42^b^	0.21	< 0.0001	< 0.0001
4-methylphenol	0.05^a^	0.54^b^	0.89^b^	0.11	< 0.0001	< 0.0001
Indole	0.00^a^	0.06^ab^	0.21^b^	0.05	0.0090	0.0254
Ammonia	65.1^a^	105.6^b^	127.3^b^	8.31	< 0.0001	< 0.0001
Fecal IgA, mg/g	16.8^b^	13.9^b^	7.9^a^	1.59	0.0002	0.0010

^1^Diets enriched in resistant starch (ERS), a fiber-prebiotic-probiotic blend (FPPB), or a fiber-prebiotic-probiotic blend + immuno-modulating ingredients (iFPPB).^2^Fecal score: 1 = hard, dry pellets; small hard mass; 2 = hard formed, dry stool; remains firm and soft; 3 = soft, formed and moist stool, retains shape; 4 = soft, unformed stool; assumes shape of container; 5 = watery, liquid that can be poured

^a,b^ Mean values within a row with unlike superscript letters differ (P < 0.05)

### Fecal characteristics and metabolites

**Fig. 1:**
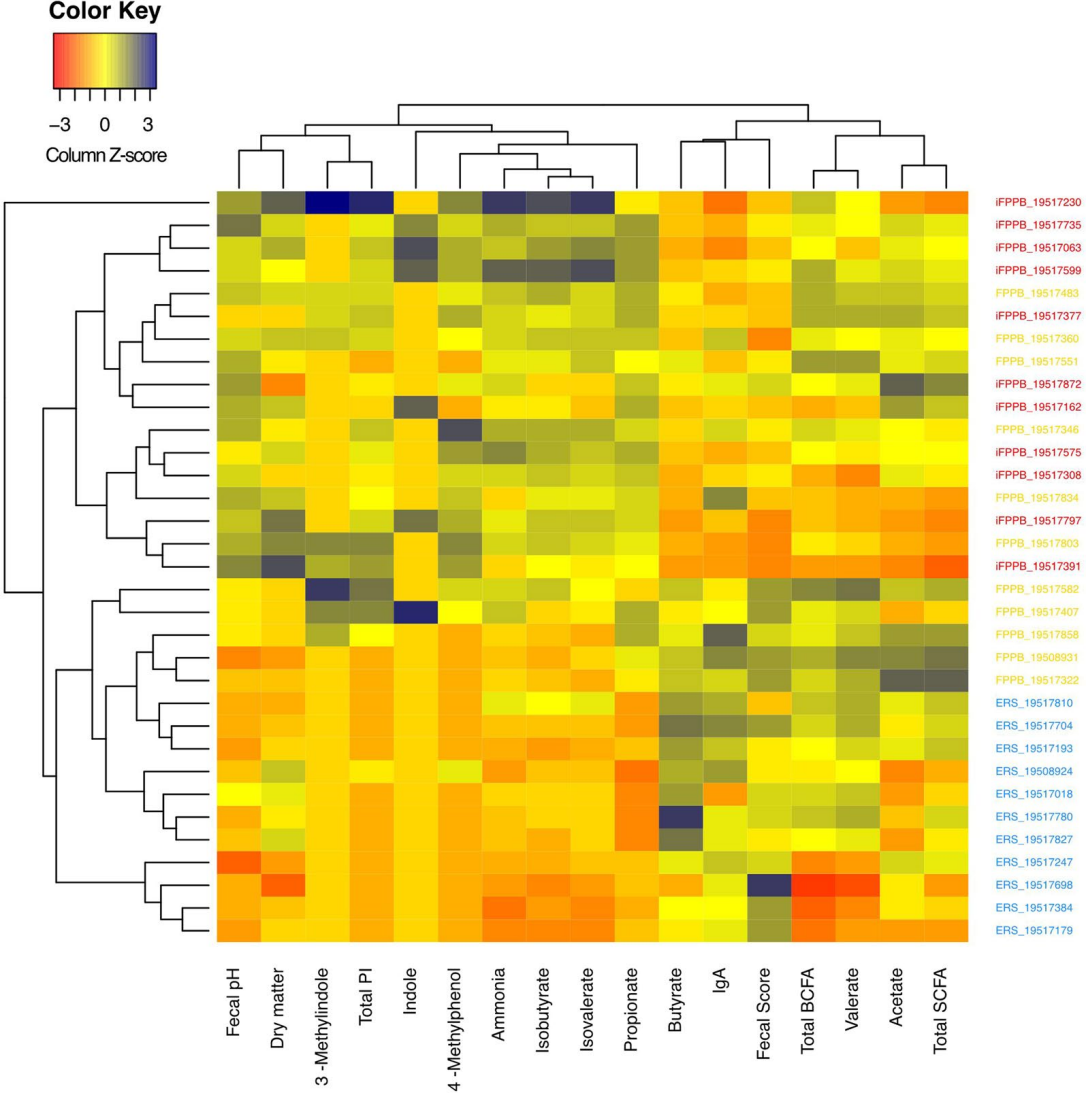
Heatmap of fecal characteristics and metabolite concentrations of cats fed the experimental diets. The data show differences among diet groups, with those fed ERS (blue text) clustering separately from those fed FPPB (yellow text) and iFPPB (red text)

Fecal characteristics and metabolite concentrations were measured to assess dietary effects on GI tolerance (i.e., fecal scores, pH, DM%) and microbial metabolism. These measurements were strongly affected by diet (Fig. 1). Fecal pH was lower (*P* < 0.05) in cats fed ERS than those fed FPPB or iFPPB (Table 1**)**. Fecal scores were lower (*P* < 0.05; firmer stools) and fecal DM % were higher (*P* < 0.05) in cats fed iFPPB than those fed ERS. Several fecal metabolites were altered by dietary treatment (Table 1). Cats fed ERS had lower (*P* < 0.05) fecal propionate, isobutyrate, isovalerate, total phenol and indole, 4-methylphenol, and ammonia concentrations than cats fed FPPB or iFPPB. Fecal butyrate concentrations also differed among treatment groups. Cats fed FPPB and iFPPB had lower (*P* < 0.05) fecal butyrate than cats fed ERS. Fecal total BCFA concentrations were lower (*P* < 0.05) in cats fed ERS than those fed FPPB. Fecal valerate concentrations were lower (*P* < 0.05) in cats fed iFPPB than those fed FPPB. Fecal indole concentrations were lower (*P* < 0.05) in cats fed ERS than those fed iFPPB. Fecal IgA concentrations were lower (*P* < 0.05) in cats fed iFPPB than those fed ERS or FPPB. In summary, fecal characteristics and metabolites were strongly affected by diet, with most of the differences being observed between the ERS diet and FPPB/iFPPB diets.

### Immune cell responsiveness to TLR agonists

To assess immune cell responsiveness, TNF-α production was measured from cells stimulated with TLR agonists (Additional file 1: Table S3). TNF-α concentrations of unstimulated control wells were not different among treatment groups. In those stimulated with Poly I:C (TLR3 agonist), TNF-α concentration was higher (*P* < 0.05) in cells from cats fed iFPPB than those fed ERS. In those stimulated with zymosan (TLR2 agonist), TNF-α concentration tended to be higher (*P* = 0.06) in cells from cats fed FPPB or iFPPB than those fed ERS. No differences were observed in cells stimulated with lipopolysaccharide (TLR4 agonist) or R848 (TLR7/8 agonist). Overall, TLR stimulation led to moderate changes in cats fed the test diets.

### Serum chemistry profile and blood cell counts

At the end of each experimental period, blood samples were collected for serum chemistry and hematology analysis to ensure that cats remained healthy throughout the study. Serum metabolites were within reference ranges for all cats, except for glucose, creatine phosphokinase and sodium to potassium ratio (Additional file 1: Table S4). Glucose was slightly higher than the reference range in cats FPPB or iFPPB, and sodium to potassium ratio was slightly higher in cats fed ERS or iFPPB. Creatine phosphokinase was higher than the reference range for all treatment groups. Blood urea nitrogen, globulin, and cholesterol concentrations were lower (*P* < 0.05) in cats fed ERS than those fed FPPB or iFPPB. Most of the blood cell counts were within the reference ranges for all cats, except for lymphocytes, monocytes, and eosinophils, which were above the reference ranges for all treatment groups (Additional file 1: Table S5). No statistically significant differences were observed among treatment groups; however, mean cell volume was higher (*P* < 0.05) in cats fed FPPB or iFPPB than those fed ERS. Overall, few serum metabolites and blood counts were affected by diet. Most were within reference ranges and cats remained healthy throughout the study; therefore, these small differences are unlikely to have physiological relevance.

### Composition of the feline gut microbiome based on shotgun metagenomic sequencing

To evaluate the effect of diet on the feline gut microbiome, we performed shotgun sequencing on a total of 33 fecal samples from the 11 cats fed each dietary treatment. Because there was a sample missing from one of the cats, microbiome assessment only included the 11 cats from which samples existed from all 3 treatments. After quality control and filtering of low-quality reads, 70,291,067 reads (average 2,130,032 ± 776,926 reads per sample) were aligned to a bacterial database, including a comprehensive list of bacterial reference genomes, and a total of 1690 bacterial species were identified (Additional file 2: Fig. S1 A–C).

**Fig. 2:**
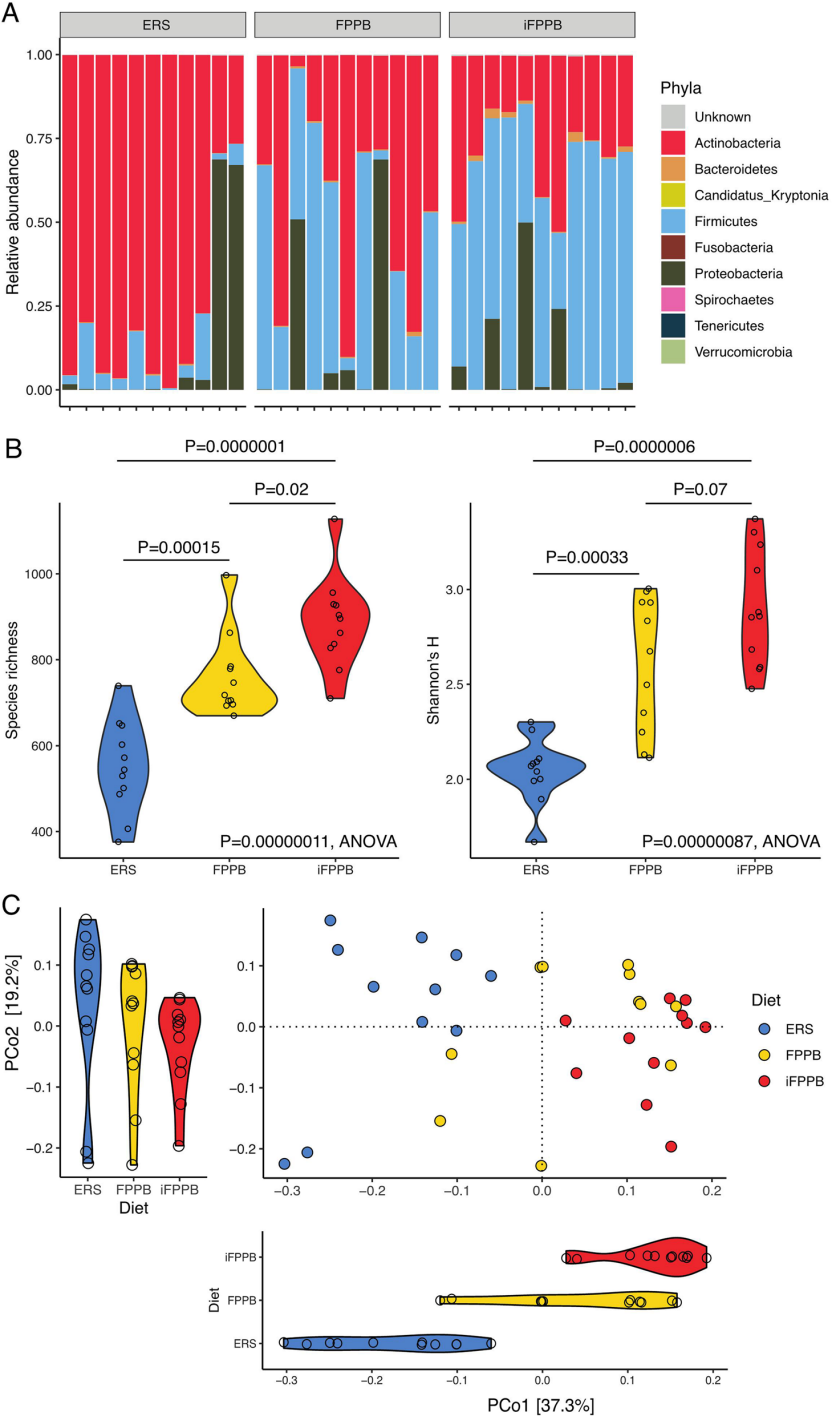
Bacterial phyla relative abundance, alpha diversity measures, and beta diversity of fecal samples collected from cats fed the experimental diets. Shifts in fecal bacteria are demonstrated among the diet groups. **A** Relative abundances at the phylum level for each dietary group. **B** Alpha diversity differences between the diet groups. **C** Principal Coordinate Analysis (PCoA) of species-level gut microbiomes using Bray-Curtis distances. PERMANOVA was used to assess the association between diet and period with the gut microbiome composition. The Kruskal–Wallis test was used to assess association between diet and the top two axes (PCo1, PCo2) followed by Dunn’s post-hoc test to evaluate the difference between the dietary groups

Three phyla were most prevalent in the gut microbiome of cats regardless of dietary treatment: Actinobacteria, Firmicutes, and Proteobacteria (Fig. 2A). In cats fed ERS, Actinobacteria was the dominant phylum (79% ± 26%), followed by Proteobacteria (13% ± 27%) and Firmicutes (8% ± 7%). The relative abundance of Actinobacteria was higher and that of Firmicutes was lower in cats fed ERS when compared with those fed FPPB or iFPPB (FDR adjusted *P* < 0.0001 and = 0.002, respectively). The relative abundance of both Actinobacteria and Firmicutes were intermediate in cats fed FPPB (47% ± 29% and 41% ± 27%, respectively), whereas the relative abundance of Firmicutes was highest (59% ± 18%) and that of Actinobacteria was lowest (30% ± 13%) in cats fed iFPPB. The relative abundance of Proteobacteria remained relatively stable regardless of diet fed (FDR adjusted *P* = 0.32).

Diet significantly affected α-diversity, as measured by species richness and Shannon’s diversity index (*P* < 0.001, ANOVA). Both measures of α-diversity were lowest in cats fed ERS, intermediate in those fed FPPB, and highest in cats fed iFPPB (*P* < 0.001, Tukey’s HSD test; Fig. 2B; Additional file 2: Fig. S2A, B). Neither of the two α-diversity measures nor the gut microbiome composition differed significantly across experimental periods (*P* = 0.25 and 0.68 for species richness and Shannon’s diversity index respectively, and *P* > 0.10, PERMANOVA for composition (Additional file 2: Fig. S2C, D).

Principal coordinates analysis revealed a noticeable shift in the gut microbiome composition among cats fed the three dietary treatments (*P* < 0.0001, PERMANOVA; Fig. 2C). The primary principal coordinate axis (PCoA1) was strongly influenced by diet [*P* < 0.0001, Kruskal–Wallis test (KWt)] and it separated cats fed ERS from those fed iFPPB (FDR adjusted *P* < 0.0001, Dunn’s post-hoc test), with those fed FPPB having an intermediate position (FDR adjusted *P* = 0.0025, Dunn’s post-hoc test). An association between diet and PCoA2 was not observed (*P* = 0.14, KWt). Similar results were also observed when using Jensen-Shannon distance to assess bacterial composition (Additional file 2: Fig. S3A–D).

### Diet-associated bacterial taxa changes

**Fig. 3:**
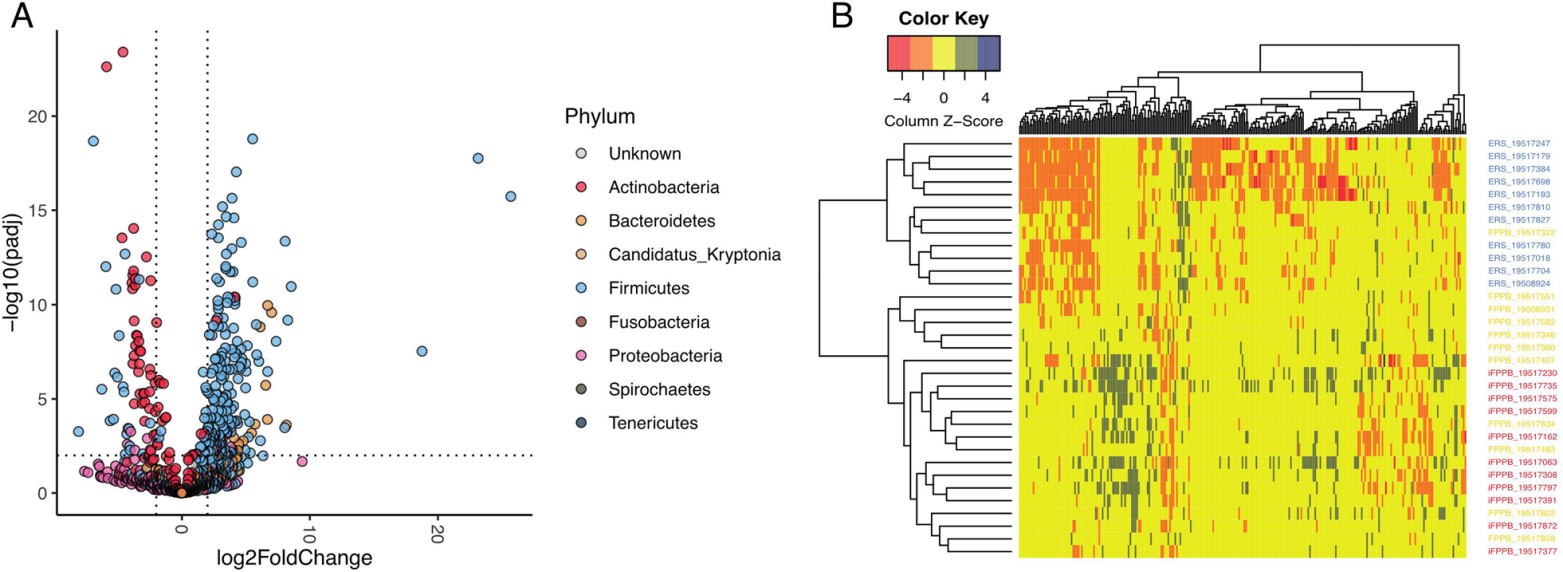
Bacterial species differences of fecal samples collected from cats fed the experimental diets. **A** A volcano plot showing differential abundance of 259 bacterial species between diets. Each dot is a bacterial species and dots are colored by phylum. Positive values in x-axis represent species that increased in abundance in the FPPB and/or iFPPB diets relative to ERS and negative values in the x-axis represent species that decreased in abundance in these diets relative to the ERS diet. The horizontal dotted line represents significance threshold of FDR adjusted *P*-value < 0.01 obtained from DESeq2 and the two horizontal lines differentiate the species with log2 fold change in abundance. Species with FDR adjusted P values < 0.01 and absolute log2 fold change > 2 were considered statistically significant. **B** Heatmap showing differences in relative abundance of differentially abundant bacterial species between the diet groups

We used DESeq2 to compare changes in read counts at the species level in cats fed FPPB and/or iFPPB relative to those fed ERS. The relative abundance of 259 bacterial species were different in cats fed FPPB and/or iFPPB compared with those fed ERS (FDR adjusted *P* < 0.01, GLM and absolute log2 fold change > 2; Fig. 3A). A total of 162 species, of which 143 species belonged to the Firmicutes phylum, had a greater relative abundance in cats fed FPPB and/or iFPPB than those fed ERS, whereas 58 species, of which 26 were from the Actinobacteria phylum, were lower in cats fed FPPB and/or iFPPB than those fed ERS (Additional file 1: Tables S6–S7). To test whether the change in relative abundance was consistent in both FPPB and iFPPB, we compared them individually with ERS and observed that most of the 259 species were also significantly differentially abundant in each comparison (Additional file 2: Fig. S4A, B). Furthermore, only 7 species were significantly different between FPPB and iFPPB (Additional file 2: Fig. S4C). Hierarchical clustering of the relative abundance of the 259 differentially abundant species showed a clear distinction between ERS and FPPB/iFPPB, although the latter did not differentiate into separate clusters (Fig. 3B).

Of the 259 differentially abundant species, 143 were in the Firmicutes phylum and included 14 species of *Blautia*, 14 species of *Clostridium*, and 5 species of *Lactobacillus* whose relative abundance was increased by FPPB/iFPPB (Additional file 1: Table S6). The FPPB/iFPPB treatments also increased the relative abundances of 13 species in the Bacteroidetes phylum. The Actinobacteria whose relative abundances were depleted by FPPB/iFPPB included 24 species of *Bifidobacterium* (Additional file 1: Table S7). Moreover, some Proteobacteria species belonging to the genera *Campylobacter* and *Helicobacter* also showed depletion in cats fed FPPB/iFPPB relative to those fed ERS.

### Bacterial gene abundance, functional modules and enzymes affected by diet

**Fig. 4:**
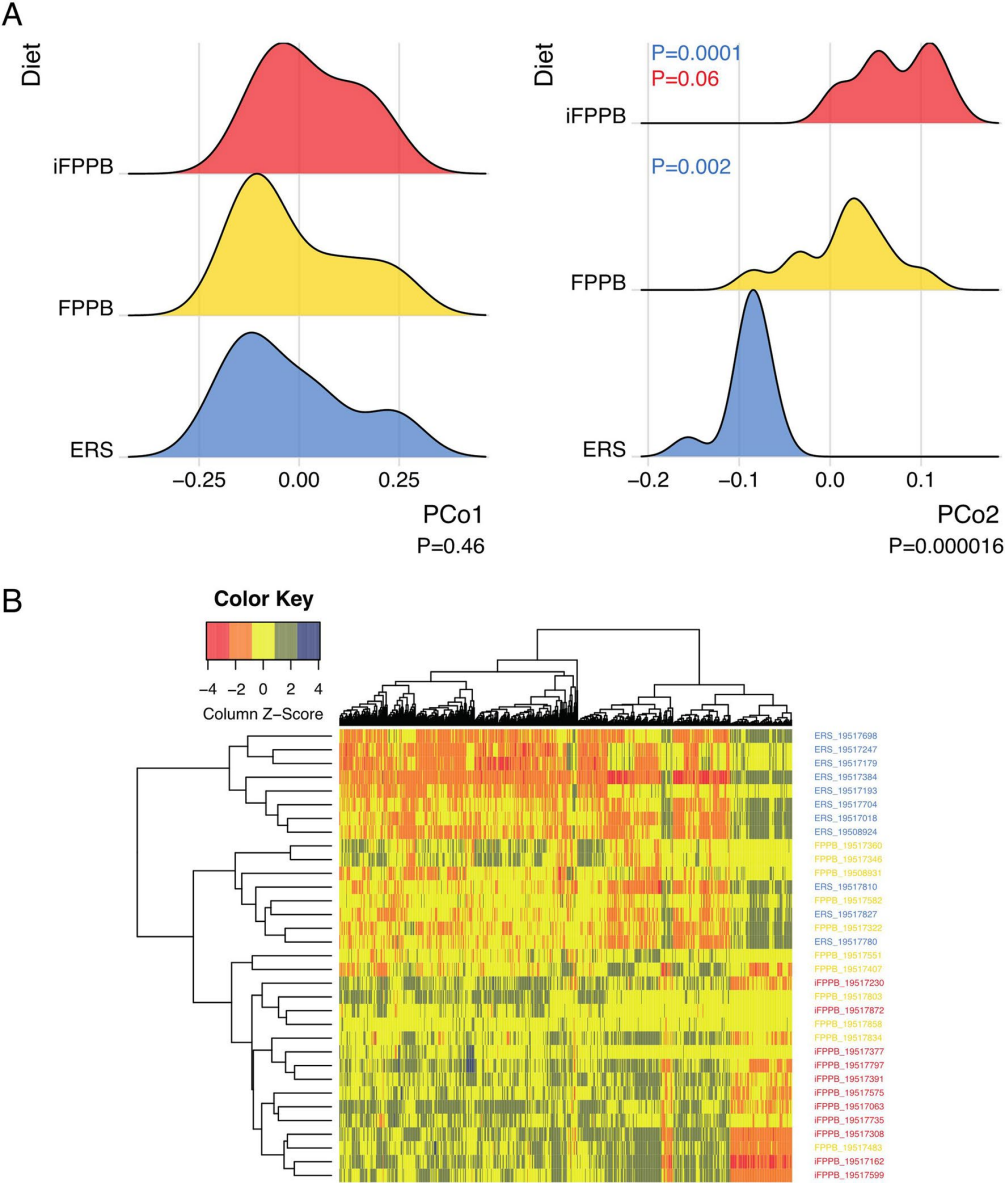
Principal coordinate analysis (PCoA) and heatmap of Kyoto Encyclopedia of Genes and Genomes (KEGG) orthology (KO) terms of fecal samples collected from cats fed the experimental diets. The data show a significant shift in KO term relative abundance between the diet groups, indicating gut bacterial functions changed in response to diet. The Kruskal–Wallis test was used to assess the association between diet and the top two PCo axes followed by Dunn’s post-hoc test to evaluate the difference between groups. **A** No significant differences between the diet groups were observed along the PCo1 (*P* > 0.05, Kruskal–Wallis test). However, strong shifts along the PCo2 was observed between the diet groups (*P* < 0.0001, Kruskal–Wallis test). PCo2 scores were higher in iFPPB and FPPB groups (FDR adjusted *P*-values = 0.0001 and 0.002, respectively, Dunn’s post-hoc test) but they did not differ significantly between the iFPPB and FPPB groups (*P* = 0.06, Dunn’s post-hoc test). **B** Heatmap showing differences in relative abundance of differentially abundant KO terms between the diet groups

To test the effects of diet on changes in microbial function, we first performed a principal coordinates analysis (PCoA) using the KO terms (Fig. 4A). The primary principal coordinate axis (PCo1) was not affected by diet (*P* = 0.46, KWt), but PCo2 showed a strong shift due to diet (*P* < 0.0001, KWt). Principal coordinate axis (PCo2) scores were higher for FPPB (FDR adjusted *P* = 0.002, Dunn’s post-hoc test) and iFPPB (FDR adjusted *P* < 0.0001, Dunn’s post-hoc test) than ERS. However, differences in PCo2 scores between FPPB and iFPPB were marginal (*P* = 0.06). A heatmap representation of the relative abundance of differentially abundant KO terms showed a separate clustering of ERS from iFPPB, while FPPB did not differentiate into a separate cluster (Fig. 4B), which is consistent with changes in bacterial relative abundances (Fig. 3B).

A total of 3624 KO terms were identified, and after filtering, 212 KO terms were identified as being differentially abundant among dietary groups using DESeq2. Of these, the relative abundances of 109 KO terms were higher in cats fed ERS compared to those fed FPPB and iFPPB (Additional file 1: Table S8). A total of 103 KO terms had higher abundances in cats fed FPPB or iFPPB relative to ERS (Additional file 1: Table S9). The primary metabolic pathways affected in all dietary treatments were those associated with carbohydrate metabolism, biosynthesis of amino acids, and metabolism of cofactors and vitamins.

### Random forest analysis of shotgun data predicts dietary shifts in bacterial taxonomy

PAM clustering using the top PCo axes (Additional file 2: Fig. S5) revealed that cats in this study could be clustered into two clusters. Cluster 1 contained cats fed iFPPB (11 cats) and most of the cats fed FPPB (8 cats), while Cluster 2 represented samples from cats fed ERS (11 cats) and a few from cats fed FPPB (3 cats). Furthermore, a random forest classification analysis on shotgun data was able to accurately differentiate the microbial composition between the three diet groups, as ERS and iFPPB cats were predicted as ERS and iFPPB with 100% (11 of 11) and 91% (10 of 11) accuracy, respectively, while the FPPB cats were split between the three groups (3 predicted as ERS, 5 predicted as FPPB, and 3 predicted as iFPPB). These results collectively provide additional evidence demonstrating a clear difference between the gut microbiomes of cats fed ERS and iFPPB.

### Diet-microbiome-metabolite relationships

**Fig. 5:**
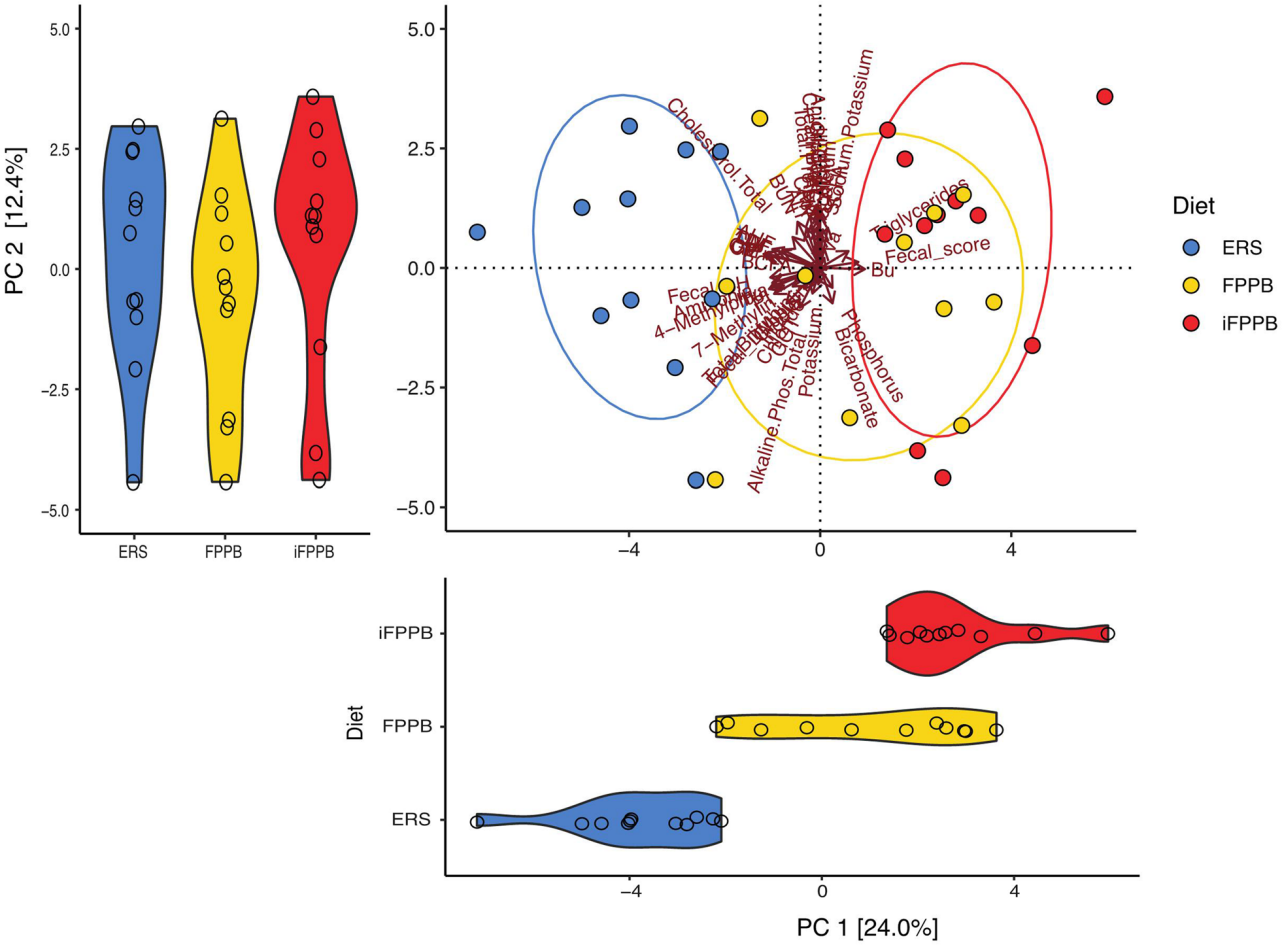
Principal component analysis of fecal and blood sample data from cats fed the experimental diets. The data show that serum triglyceride concentrations, fecal butyrate concentrations, and fecal scores were the primary variables driving principal component 1 in cats fed FPPB and iFPPB and fecal pH and fecal ammonia concentrations were the primary variables driving principal component 1 in cats fed ERS. Each dot represents a single cat eating one of the experimental diets

The effects of diet on fecal metabolites showed a separate clustering of cats fed ERS from those fed iFPPB, with those fed FPPB being intermediate but more similar to iFPPB (Fig. 5). Principal component analysis (PCA) indicated that principal component (PC) 1 and 2 explained 24.0% and 12.4% of variability respectively, and PC1 score for ERS was lower (*P* < 0.05) than that of FPPB and iFPPB (Fig. 5). Some of the main metabolites driving the increased PC1 values in FPPB and iFPPB were serum triglyceride concentrations, fecal butyrate concentrations, and fecal scores. On the other hand, fecal pH and fecal ammonia concentrations are some of the primary factors that decreased PC1 values in ERS. Diet did not have a significant effect on PC2 scores.

**Fig. 6:**
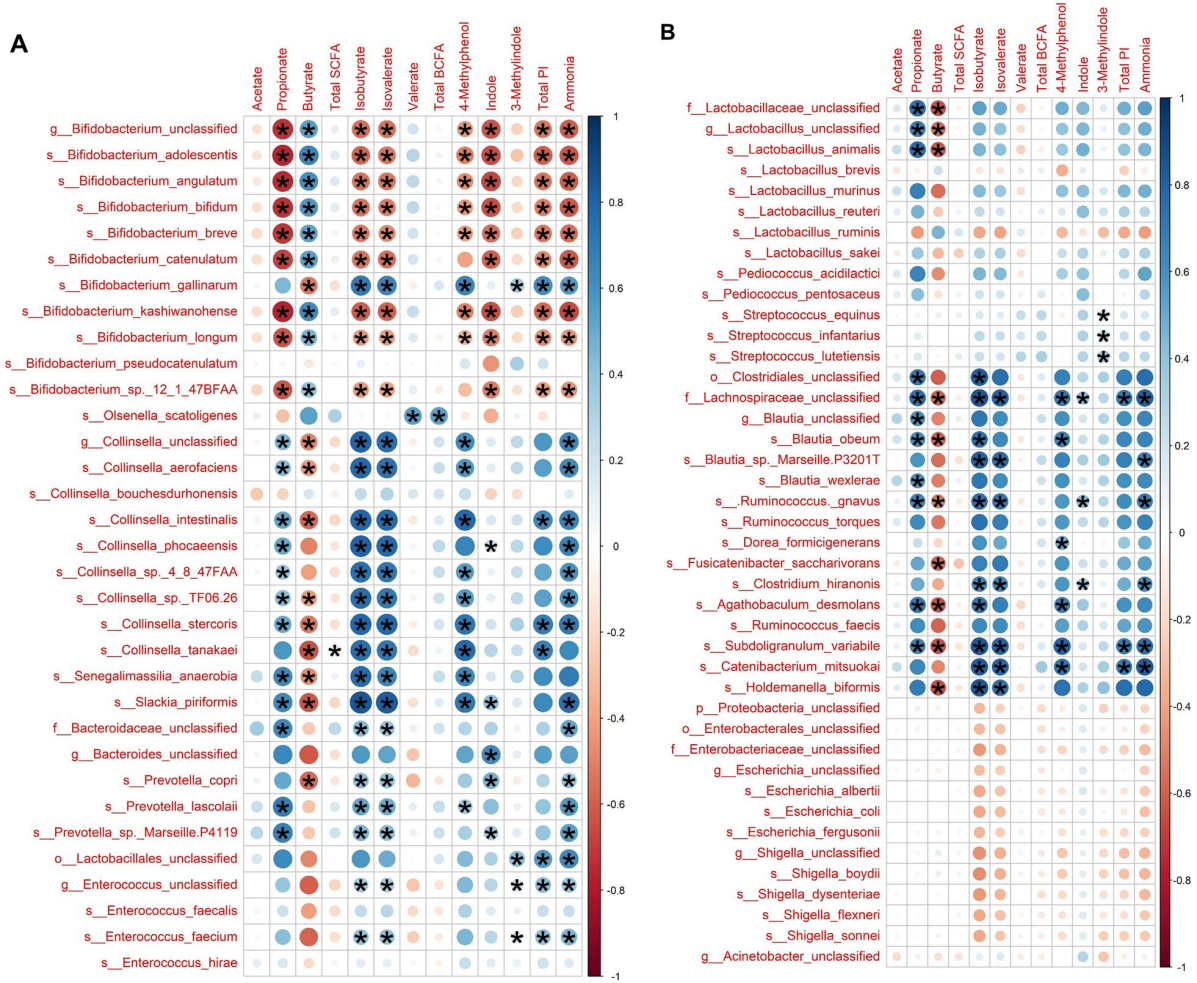
Correlation plots between fecal microbial species and fecal metabolites of cats fed the experimental diets. The X and Y axes are the metabolites and species, respectively. R values are represented by different colors (blue: positive; red: negative). Significant correlations (*P* adj < 0.05) are indicated by *

Correlations between fecal microbiome measures and fecal metabolite concentrations were evaluated using Spearman’s rank correlation test (Fig. 6A, B). In general, the relative abundances of *Bifidobacterium* spp. were negatively correlated (*P* < 0.05) with fecal propionate, isobutyrate, isovalerate, 4-methylphenol, indole, total phenol and indole (total phenols and indoles) and ammonia concentrations, while they were positively correlated (*P* < 0.05) with fecal butyrate concentrations. Most *Collinsella* spp. relative abundances were positively correlated (*P* < 0.05) with fecal propionate, isobutyrate, isovalerate, 4-methylphenol and ammonia concentrations, while being negatively correlated (*P* < 0.05) with fecal butyrate concentrations. The relative abundances of most *Lactobacillus* spp. were positively correlated (*P* < 0.05) with fecal propionate, but negatively correlated (*P* < 0.05) with fecal butyrate concentrations. Lastly, the relative abundances of *Blautia*, Lachnospiraceae, and *Ruminococcus* spp. were positively correlated (*P* < 0.05) with fecal propionate, isobutyrate, isovalerate, ammonia, and total phenol and indole concentrations, but negatively correlated (*P* < 0.05) with fecal butyrate concentrations.

## Discussion

Functional ingredients targeting gastrointestinal health are increasingly popular dietary inclusions, and may include dietary fibers, prebiotics, probiotics or postbiotics added to commercial pet foods or sold separately as supplements. In addition to GI health, yeast-based ingredients and SDAP are often marketed as improving overall ‘immune health.’ When considering impacts on potential markers of GI health, many studies have focused on the effects of diet on gut microbiota utilizing 16 S rRNA gene microbial profiling methods. That strategy provides some insights into gut microbial ecology, but often only characterizes bacteria to the genus level. Strategies allowing for a deeper resolution (i.e., to the species level) and the characterization of microbial gene content, which provides more information on the functionality and metabolic potential, are needed to expand our understanding of these populations and how they interact with and influence the host. To address these needs, the primary objective of the current study was to characterize and report the effect of dietary changes on the fecal metagenome and metabolite profiles of healthy adult cats fed diets enriched in RS or dietary fibers and a biotics mixture. Another objective was to identify significant bacterial taxa-bacterial gene-metabolite correlations observed in these cats. Lastly, the effects of diet on the apparent total tract macronutrient digestibility, stool quality and characteristics, fecal fermentative metabolites, and immune indices of the cats were evaluated.

Dietary macronutrient profile (e.g., protein: carbohydrate ratio), dietary fiber amount and type, and the form of food consumed (e.g., raw vs. extruded diets) have been previously shown to alter the feline GI microbiome [38, 39]. In humans, the dietary protein to carbohydrate ratio quickly and dramatically impacts the GI microbiome [40]. Similar dietary shifts alter the GI microbiome and blood metabolome of cats [3, 41, 42]. Dietary fibers, prebiotics, and RS also influence stool characteristics and gastrointestinal microbiota in cats [1, 43–45]. Yeast-based functional ingredients have been tested in dogs and shown to alter GI microbiota and/or markers of immune function [16, 46, 47]. SDP is another immune modulator that has been shown to attenuate innate immunity, intestinal barrier function, and reduce intestinal inflammation in rodent models [11, 48, 49]. Even though SDP is known to be highly palatable and digestible by dogs and cats [50, 51], it has not been tested for its impact on GI microbiota and immune function in these species.

Non-digestible carbohydrates have been shown to alter aspects of feline metabolism, including altered glucose and amino acid metabolism, and a reduction in uremic toxins [52–54]. RS is also known to modulate microbiota activity, most notably by increasing butyrate production [55, 56]. Although the effects of RS have been extensively studied in humans and rodent models [57], there are only a few examples in dogs [56, 58, 59], and its effects on obligate carnivores have not been well studied. The consumption of RS has been associated with increased fecal SCFA and is known to lead to greater butyrate production in cats [60]. This agrees with the increased fecal butyrate observed in cats fed the ERS diet in the current study, which was likely due to the poorly-gelatinized potato starch reaching and being fermented in the colon. The fecal SCFA concentrations for cats in all treatments were rather high compared with previous studies, which was likely due to the relatively high dietary fiber concentrations and low nutrient digestibilities that would provide plenty of fermentable substrate to the microbiota in the colon. In a human study, *Bifidobacterium faecale*/*adolescentis*/*stercoris* and *Ruminococcus bromii* were recognized as being the primary microbial species driving potato starch degradation [61–63]. The data in the current study agrees, with the relative abundance of *B. adolescentis* and many other *Bifidobacterium* spp. increasing in cats fed the ERS diet compared to the other diet groups. Consumption of RS has also been associated with increased GI IgA concentrations, which are thought to be a butyrate-induced response. This response has been observed in many different mammalian species, including cats in a previous study [60] and those in the current study.

Although many of the functions of microbial genes and/or activity of bacterial species in the GI tract remain uncharacterized, decades of traditional culture methods and recent microbiome research has shed light on many of the predominant taxa in the GI tract. Firmicutes, for instance, is known to have many carbohydrate fermenters and SCFA producers that ferment dietary fibers and other non-digestible carbohydrates. Many of these bacterial taxa were greater in cats fed the FPPB and iFPPB diets, with corresponding SCFA (acetate; propionate) also being greater. The elevated relative abundance of some genera in the Firmicutes phylum (i.e., *Lactobacillus* spp.; *Pediococcus* spp.) were of particular interest in the specialized diets, as these taxa are known to regulate fermentation and immune responses in mice [64]. In addition, the relative abundance of *Bacteroides vulgatus* (name recently changed to *Phocaeicola vulgatus*), which has been shown to reduce liposaccharide in the GI tract, was elevated in cats fed the FPPB or iFPPB diets [65]. *Blautia obeum*, which is known to produce a lantibiotic peptide, effective against multiple *Clostridium* species and to hydrolyze bile salts, was elevated in cats fed the FPPB or iFPPB diets [66–68]. In humans, *Holdemanella biformis* is recognized as a bacterium that reduces tumor cell proliferation and was reported to be greater in cats fed FPPB or iFPPB diets [69]. The relative abundance of *O. scatoligenes* in the Actinobacteria phylum was depleted in cats fed the FPPB or iFPPB diets relative to ERS. This taxon is known to be a skatole and p-cresol producer [70]. Because fecal phenol and indole concentrations were greater in cats fed FPPB or iFPPB, other bacterial taxa must have contributed to this process.

Enrichment of Actinobacteria, particularly *Bifidobacterium* spp., was observed in the ERS group. *Bifidobacterium* spp. are a commensal bacteria that have been extensively studied in humans for their beneficial effects in treating and preventing GI and immune diseases [71]. Although *Bifidobacterium* spp. are carbohydrate fermenters and some (i.e., *B. choerinum*, *B. longum*, *B. pseudolongum, B. adolescentis*) are known to hydrolyze RS [72–75], not all subspecies have that capability. *B. longum* has been extensively studied in human and mice and recognized for its effect in attenuating and preventing the inflammatory bowel diseases, ulcerative colitis and Crohn’s disease. This bacterial species enhances antioxidant activity to regulate oxidative stress levels, helping attenuate intestinal inflammatory response in mice with experimentally induced colitis model [76]. It also enhances intestinal barrier function by increased epithelial barrier function, and in humans, it has been shown to decrease depression scores [77, 78]. *Corynebacterium pyruviciproducens* was also enriched in cats fed ERS. This Actinobacteria member is a relatively novel species and known pyruvate producer that has been isolated from different parts of the mammalian body [79]. In vitro studies using human cells have suggested that it acts as an immunoadjuvant by promoting a humoral immune response to pathogens [80]. *Campylobacter* spp., *Helicobacter* spp. and *Pseudomonas* spp. were enriched in the ERS group, which may have been due to greater protein fermentation in the gut. Although the ERS diet contained the lowest crude protein concentrations, cats fed that diet also had a much lower protein digestibility. In many different mammalian species, these Proteobacteria members are linked with infectious GI diseases [81]. Because many of these strains have been isolated from the feces of healthy dogs and cats, their presence does not guarantee disease, but they may serve as opportunistic pathogens for the pets or their owners [82, 83]. Many of these bacterial species have been characterized in culture or in other host species, but have been poorly studied in cats. Therefore, more studies dedicated to understanding how changes in specific microbial populations affect the health of cats are needed.

Although KO term abundances were different among cats fed ERS, FPPB, and iFPPB diets, all diets had an enrichment of genes associated with carbohydrate metabolism, biosynthesis of amino acids, and metabolism of cofactors and vitamins. These results show that although changes in the bacterial community may occur and they may be somewhat predictable between ERS, FPPB, and iFPPB diets, there is a large overlap in terms of functional capacity and metabolic potential. Given the complex nature of the test diets in this study, with each containing different amounts and/or sources of dietary fiber, probiotic, RS, and yeast product, it would be difficult to attribute changes at the KO term level to any of the ingredients. Future studies that test diets differing in a single or a small number of ingredients/nutrients would be needed to make such connections.

In addition to changes in the microbial community, physiological outcomes and immune responses were also measured. All hematological measurements were within ranges for healthy adult cats, with only blood urea nitrogen and cholesterol being lower in cats fed the ERS diet than those fed the FPPB or iFPPB diets which is of questionable clinical significance. The lower blood cholesterol concentrations in ERS-fed cats agrees with other research demonstrating blood-lipid lowering effect of RS in mice fed high-fat diets [84]. RS has also been shown to exert a blood urea-lowering effect by enhancing urea nitrogen transfer in rats [85]. From the measured immune responses, only cells stimulated with Poly I:C were different among groups, with TNF-α being elevated in cells from cats fed the iFPPB diet compared to those coming from cats fed the ERS diet. Poly I:C is a TLR3 agonist, which recognizes dsRNA of viral origin. More research is needed to determine whether the functional ingredients contained in the iFPPB diet enhances response to viral challenge. Overall, small physiological changes were observed throughout the study and all cats remained healthy.

## Conclusion

In summary, this study demonstrated that diets containing different dietary fibers and biotic ingredients affect the fecal microbial diversity, metabolite composition, and microbial gene content in cats. By utilizing shotgun-based metagenomic sequencing, we identified striking differences among diets fed. In addition to changes in fecal bacterial alpha diversity, a large number of bacterial taxa were shown to shift due to diet. As expected, increased fecal butyrate and IgA concentrations, but reduced fecal BCFA concentrations were observed in cats fed the high-RS diet. To our knowledge, this is one of the first studies utilizing shotgun sequencing technology to study the microbiome of the feline GI tract. Future studies should evaluate diets with fewer macronutrient and ingredient differences so that specific taxa/gene responses may be identified.

## Supplementary information

**Additional file 1**:** Table S1**. Ingredient and analyzed nutrient composition of the experimental diets fed to cats.** Table S2**. Body weight, BCS, and food intake of cats fed the experimental diets.** Table S3**. TNF-α concentrations (ng/L) of cell culture supernatants from cats fed the experimental diets.** Table S4**. Serum chemistry profiles of cats fed the experimental diets.** Table S5**. Hematology of cats fed the experimental diets.** Table S6**. Bacterial species that were greater in feces of cats fed FPPB or iFPPB than those fed ERS.** Table S7**. Bacterial species that were lower in feces of cats fed FPPB or iFPPB than those fed ERS.** Table S8**. Kyoto Encyclopedia of Genes and Genomes Orthology (KO) terms that were greater in cats fed ERS than those fed FPPB or iFPPB. **Table S9**. Kyoto Encyclopedia of Genes and Genomes Orthology (KO) terms that were greater in cats fed FPPB or iFPPB than those fed ERS.

**Additional file 2**:** Fig. S1**. Quality control measures of the microbiome data. (A) Sequencing coverage of bacterial taxa and samples in the dataset before quality control. Each column is a taxa of a sample. (B) Sequencing coverage of bacterial taxa and samples after removing rare taxa present in less than 5% of the samples. Each column is a taxa or a sample. (C) Abundance (x-axis) and prevalence (y-axis) of bacterial taxa in the dataset after quality control. Each dot is a taxa and they are faceted by phylum.** Fig. S2**. Rarefaction curves of fecal samples collected from cats fed the experimental diets. The curves show alpha diversity differences between diet groups. (A, B) Differences in Species Richness and Shannon’s diversity index (Shannon’s H) between the three diet groups. (C, D) No difference in these measures were observed between the periods during which the different diets were administered.** Fig. S3**. Fecal microbial composition of cats fed the experimental diets. (A) Screeplot showing the eigenvalues obtained from PCoA using Bray-Curtis (left) and Jensen-Shannon distances (right). (B-D) Principal Coordinate Analysis (PCoA) of species-level gut microbiomes using Jensen-Shannon distances. PERMANOVA was used to assess the association between diet and period with the gut microbiome composition. Kruskal-Wall is test was used to assess association between diet and the top two axes (PCo1, PCo2) followed by Dunn’s post-hoc test to evaluate the difference between the dietary groups. (B) A PCoA plot using Jensen-Shannon distance revealed significant association between diet groups (P = 0.0001, PERMANOVA). Period was also significant in this case but with much weaker significance (P = 0.034, PERMANOVA). (C) No difference in PCo1 or PCo2 between the periods (P = 0.12 and 0.41, respectively, Kruskal–Wallis test). (D) Diet was associated with both PCo1 and PCo2 (P < 0.0001 and 0.03, respectively, Kruskal Wallis test).** Fig. S4**. Volcano plots of fecal microbiota data from cats fed the experimental diets. The plots show differential abundance of bacterial species between diets. Each dot is a bacterial species and dots are colored by phylum. Positive values in x-axis represent species that increased in abundance in the FPPB and/or iFPPB diets relative to ERS and negative values in the x-axis represent species that decreased in abundance in these diets relative to ERS. The horizontal dotted line represents significance threshold of FDR adjusted P-value < 0.01 obtained from DESeq2 and the two horizontal lines differentiate the species with log2 fold change in abundance. Species with FDR adjusted P values < 0.01 and absolute log2 fold change > 2 were considered statistically significant. ERS vs FPPB only (A), ERS vs iFPPB only (B). Comparison of FPPB vs iFPPB revealed only 7 of differentially abundant species (C).** Fig. S5**. Partition Around Medoids clustering analysis of fecal microbiota data from cats fed the experimental diets. The analysis revealed two clusters, with Cluster 1 containing primarily FPPB and iFPPB diet groups and Cluster 2 containing primarily the ERS group.

## Data Availability

All sequence data are available at the NCBI sequence read archive under BioProject PRJNA906124.

## Abbreviations

BCS: Body condition score
FDR: False discovery rate
KEGG: Kyoto encyclopedia of genes and genomes
KO: KEGG orthology
KWt: Kruskal–Wallis test
OTU: Operational taxonomic unit
PBMC: Peripheral blood mononuclear cells
PCoA: Principal coordinate analysis
PERMANOVA: Permutational multivariate analysis of variance
RS: Resistant starch
SDP: Spray-dried plasma
SEM: Standard error of the mean
