# Supplementary material for: Dietary enrichment of resistant starches or fibers differentially alter the feline fecal microbiome and metabolite profile
Source: Anim Microbiome. 2022 Dec 5;4:61. doi: 10.1186/s42523-022-00213-9 (PMC9720964; doi:10.1186/s42523-022-00213-9)
Supplement: Supplementary file 1 — Additional file 1: Table S1. Ingredient and analyzed nutrient composition of the experimental diets fed to cats. Table S2. Body weight, BCS, and food intake of cats fed the experimental diets. Table S3. TNF-α concentrations (ng/L) of cell culture supernatants from cats fed the experimental diets. Table S4. Serum chemistry profiles of cats fed the experimental diets. Table S5. Hematology of cats fed the experimental diets. Table S6. Bacterial species that were greater in feces of cats fed FPPB or iFPPB than those fed ERS. Table S7. Bacterial species that were lower in feces of cats fed FPPB or iFPPB than those fed ERS. Table S8. Kyoto Encyclopedia of Genes and Genomes Orthology (KO) terms that were greater in cats fed ERS than those fed FPPB or iFPPB. Table S9. Kyoto Encyclopedia of Genes and Genomes Orthology (KO) terms that were greater in cats fed FPPB or iFPPB than those fed ERS. [file 42523_2022_213_MOESM1_ESM.docx]

| **Supplementary Table 1.** Ingredient and analyzed nutrient composition of the experimental diets fed to cats | | | |
| --- | --- | --- | --- |
| Ingredient | ERS^1^ | FPPB | iFPPB |
|  | ---------- %, as-is ---------- | | |
| Potato flour | 21.00 | --- | --- |
| Chicken meal (regular ash) | 20.00 | --- | --- |
| Menhaden fish meal | --- | 20.00 | 20.00 |
| Salmon slurry | 20.00 | 20.00 | 20.00 |
| Brewers rice | 20.58 | --- | --- |
| Oat groats | --- | 20.00 | 16.66 |
| Dried green peas | --- | 12.91 | 12.00 |
| Chicken fat (with 5000 ppm Naturox^2^) | 9.11 | 6.00 | 6.00 |
| Dried egg product | --- | 5.00 | 5.00 |
| Dried plain beet pulp | --- | 3.00 | 4.00 |
| Flaxseed meal | --- | 3.00 | 3.00 |
| Pea Fiber | --- | 3.00 | 3.00 |
| Spray dried plasma^3^ | --- | --- | 3.00 |
| Probiotic^4^ | --- | 2.30 | 2.30 |
| Dried salmon | 5.00 | 2.00 | 2.00 |
| Cat diet palatant^5^ | 2.00 | --- | --- |
| Dried brewer’s yeast | 0.92 | 0.92 | 0.92 |
| Menhaden fish oil | --- | 0.50 | 0.50 |
| Vitamin premix^6^ | 0.35 | 0.35 | 0.35 |
| Yeast fermentation product^7^ | --- | --- | 0.25 |
| Sodium hexametaphosphate | 0.20 | 0.20 | 0.20 |
| Taurine | 0.20 | 0.20 | 0.20 |
| Potassium chloride | 0.19 | 0.19 | 0.19 |
| Inulin | --- | 0.10 | 0.10 |
| Mineral premix^8^ | 0.10 | 0.10 | 0.10 |
| Choline chloride (70%, dry) | 0.10 | 0.10 | 0.10 |
| Dry antioxidant^9^ | 0.05 | 0.05 | 0.05 |
| Vitamin E (50 %, dry) | --- | 0.03 | 0.03 |
| Turmeric powder | --- | 0.03 | 0.03 |
| Stay C-35 (ascorbic acid) | --- | 0.03 | 0.03 |
|  |  |  |  |
| Chemical composition |  |  |  |
| Dry matter, % | 90.6 | 94.2 | 95.9 |
|  | --------------- %, DM --------------- | | |
| Crude protein | 21.9 | 28.5 | 31.6 |
| Acid hydrolyzed fat | 9.0 | 14.1 | 16.9 |
| Ash | 6.3 | 8.3 | 8.5 |
| Total dietary fiber | 11.2 | 12.6 | 16.7 |
| Insoluble fiber | 6.1 | 9.4 | 11.4 |
| Soluble fiber | 5.1 | 3.2 | 5.3 |
| Metabolizable energy, kcal/g | 3.30 | 3.24 | 3.34 |
| Total starch | 50.4 | 29.9 | 22.7 |
| Gelatinized starch | 35.7 | 26.3 | 21.7 |
| ^1^Diets enriched in resistant starch (ERS), a fiber-prebiotic-probiotic blend (FPPB), or a fiber-prebiotic-probiotic blend + immuno-modulating ingredients (iFPPB).  ^2^Naturox® NC Premium Liquid Antioxidant [mixed tocopherols (preservative), lecithin and rosemary extract), Kemin Industries, Des Moines, IA.  ^3^Hydrolyzed AP 700; APC Inc., Ankeny, IA.  ^4^LACTO-SACC: yeast culture (live *Saccharomyces cerevisiae* grown on media of ground yellow corn, diastatic malt and cane molasses), dried *Lactobacillus acidophilus* fermentation product, dried *Enterococcus faecium* fermentation product, dried *Aspergillus oryzae* fermentation extract, dried *Trichoderma longibrachiatum* fermentation extract, and dried *Bacillus subtilis* fermentation extract; Alltech, Nicholasville, KY.  ^5^AFB international; St. Charles, MO.  ^6^Vitamin premix: taurine, vitamin E supplement, niacin supplement, vitamin A supplement, thiamine mononitrate, pyridoxine hydrochloride, d-calcium pantothenate, riboflavin supplement, biotin, vitamin B12 supplement, menadione sodium bisulfite complex (source of vitamin K activity), calcium iodate, folic acid, vitamin D3 supplement.  ^7^TruMune; Diamond V, Cedar Rapids, IA.  ^8^Mineral premix: potassium chloride, zinc sulfate, zinc proteinate, iron proteinate, ferrous sulfate, copper proteinate, manganese sulfate, sodium selenite, manganese proteinate, copper sulfate.  ^9^Dry Naturox antioxidant [mixed tocopherols and citric acid (preservative), rosemary extract]; Kemin Industries, Des Moines, IA. | | | |

| **Supplementary Table 2.** Body weight, BCS, and food intake of cats fed the experimental diets^1^ | | | | | | |  |
| --- | --- | --- | --- | --- | --- | --- | --- |
|  | Dietary treatment | | |  | P value | | |
|  | **ERS^2^** | **FPPB** | **iFPPB** | SEM | Treatment | ERS vs. FPPB and iFPPB | |
| Body weight, kg | 3.78^a^ | 3.87^b^ | 3.83^ab^ | 0.29 | 0.0025 | 0.0014 | |
| Body condition score^3^ | 6.4 | 6.5 | 6.6 | 0.49 | 0.4557 | 0.3609 | |
| Daily intake, g/day | 53.6^a^ | 57.9^ab^ | 61.6^b^ | 3.52 | 0.0010 | 0.0008 | |
| Daily intake, kcal ME/day | 199.7^a^ | 227.0^b^ | 249.5^c^ | 13.84 | <0.0001 | <0.0001 | |
| ^1^Data represent mean data over the 28-day experimental period.  ^2^Diets enriched in resistant starch (ERS), a fiber-prebiotic-probiotic blend (FPPB), or a fiber-prebiotic-probiotic blend + immuno-modulating ingredients (iFPPB).  ^3^Nine-point body condition score system was used (Laflamme, 1997). Using this system, a score of 5 indicates a healthy body condition. | | | | | | | |

| **Supplementary Table 3.** TNF-α concentrations (ng/L) of cell culture supernatants from cats fed the experimental diets | | | | | | | | |
| --- | --- | --- | --- | --- | --- | --- | --- | --- |
|  | Dietary treatment | | |  |  | P value | | |
|  | ERS^1^ | FPPB | iFPPB | SEM | Treatment | | ERS vs. FPPB and iFPPB | |
| Control | 18.09 | 16.97 | 17.56 | 0.68 | 0.51 | | 0.33 | |
| Zymosan | 14.36 | 17.18 | 15.42 | 0.81 | 0.06 | | 0.06 | |
| Poly (I:C)^2^ | 17.89^a^ | 19.79^ab^ | 20.42^b^ | 0.71 | 0.05 | | 0.02 | |
| Lipopolysaccharide | 18.15 | 18.12 | 20.62 | 0.84 | 0.07 | | 0.25 | |
| R848 (requisimod) | 15.96 | 17.84 | 18.02 | 1.09 | 0.35 | | 0.15 | |
| ^1^Diets enriched in resistant starch (ERS), a fiber-prebiotic-probiotic blend (FPPB), or a fiber-prebiotic-probiotic blend + immuno-modulating ingredients (iFPPB).  ^2^Poly (I:C): polyinosinic:polycytidylic acid. | | | | | | | |  |

| **Supplementary Table 4.** Serum chemistry profiles of cats fed the experimental diets | | | | | | | | |
| --- | --- | --- | --- | --- | --- | --- | --- | --- |
|  |  | | Dietary treatment | | |  | P value | |
| Item | | Reference ranges^2^ | ERS^1^ | FPPB | iFPPB | SEM | Treatment | ERS vs. FPPB and iFPPB |
| Creatinine, mg/dL | | 0.4-1.6 | 1.06 | 1.07 | 1.01 | 0.068 | 0.5524 | 0.6923 |
| BUN^3^, mg/dL | | 18-38 | 18.53^a^ | 20.85^b^ | 21.02^b^ | 1.304 | 0.0165 | 0.0048 |
| Total protein, g/dL | | 5.8-8.0 | 6.73 | 7.04 | 7.03 | 0.149 | 0.1350 | 0.0485 |
| Albumin, g/dL | | 2.8-4.1 | 2.93 | 2.95 | 2.94 | 0.088 | 0.7891 | 3.1021 |
| Globulin, g/dL | | 2.6-5.1 | 3.81 | 4.09 | 4.09 | 0.123 | 0.0518 | 0.0166 |
| Albumin:globulin | | 0.6-1.1 | 0.77 | 0.72 | 0.74 | 0.032 | 0.1587 | 0.0680 |
| Calcium, mg/dL | | 8.8-10.2 | 9.12 | 9.05 | 8.98 | 0.117 | 0.2541 | 0.1477 |
| Phosphorus, mg/dL | | 3.2-5.3 | 7.99 | 5.17 | 4.97 | 2.212 | 0.5325 | 0.2682 |
| Sodium, mmol/L | | 145-157 | 149.38 | 149.09 | 148.18 | 0.595 | 0.3423 | 0.2995 |
| Potassium, mmol/L | | 3.6-5.3 | 4.08 | 4.29 | 4.07 | 0.092 | 0.1842 | 0.3467 |
| Sodium:potassium | | 28-36 | 37.00 | 34.82 | 36.64 | 0.902 | 0.1705 | 0.2146 |
| Chloride, mmol/L | | 109-126 | 115.75 | 115.11 | 115.58 | 0.571 | 0.6743 | 0.5073 |
| Glucose, mg/dL | | 60-122 | 107.33 | 127.34 | 130.25 | 17.060 | 0.4437 | 0.2313 |
| ALP^3^, U/L | | 10-85 | 23.89 | 18.09 | 27.99 | 5.401 | 0.1305 | 0.1524 |
| ALT^3^, U/L | | 14-71 | 56.31 | 64.17 | 56.31 | 6.353 | 0.0727 | 0.1793 |
| Total bilirubin, mg/dL | | 0.0-0.3 | 0.10 | 0.11 | 0.099 | 0.005 | 0.3608 | 0.4470 |
| CPK^3^, U/L | | 10-250 | 421.38 | 314.45 | 403.55 | 109.263 | 0.8998 | 0.9842 |
| Cholesterol, mg/dL | | 66-160 | 104.39^a^ | 133.10^b^ | 143.74^b^ | 8.819 | <0.0001 | <0.0001 |
| Triglycerides, mg/dL | | 21-166 | 31.68 | 28.83 | 25.49 | 2.191 | 0.0679 | 0.0459 |
| ^1^Diets enriched in resistant starch (HRS), a fiber-prebiotic-probiotic blend (FPPB), or a fiber-prebiotic-probiotic blend + immuno-modulating ingredients (iFPPB).  ^2^Reference ranges were provided from the University of Illinois Veterinary Diagnostic Laboratory.  ^3^BUN: blood urea nitrogen; ALP: total alkaline phosphatase; ALT: alanine aminotransferase; CPK: creatine phosphokinase. | | | | | | | | |

| **Supplementary Table 5.** Hematology of cats fed the experimental diets | | | | | | | | |
| --- | --- | --- | --- | --- | --- | --- | --- | --- |
|  |  | | Dietary treatment | | |  | P value | |
| Item | | Reference ranges^1^ | ERS^1^ | FPPB | iFPPB | SEM | Treatment | ERS vs. FPPB and iFPPB |
| Red blood cell | | 5.0-10.0 | 8.06 | 8.28 | 7.70 | 0.290 | 0.2938 | 0.8269 |
| Hemoglobin | | 8.0-15.0 | 10.96 | 11.46 | 10.71 | 0.474 | 0.4220 | 0.8071 |
| Hematocrit | | 30.0-45.0 | 32.03 | 33.40 | 31.19 | 1.358 | 0.3923 | 0.8513 |
| Mean cell volume | | 37.0-55.0 | 39.64 | 40.33 | 40.38 | 0.658 | 0.0933 | 0.0348 |
| White blood cell, 10^6^/µL | | 5.5-19.5 | 12.94 | 13.37 | 11.88 | 1.051 | 0.2191 | 0.6925 |
| Lymphocyte, 10^3^/µL | | 1.7-7.0 | 2.15 | 2.41 | 2.07 | 0.356 | 0.6462 | 0.7711 |
| Monocyte, 10^3^/µL | | 0.0-0.9 | 0.34 | 0.39 | 0.39 | 0.079 | 0.8790 | 0.6191 |
| Eosinophil, 10^3^/µL | | 0.0-0.8 | 0.60 | 0.74 | 0.73 | 0.151 | 0.7323 | 0.4352 |
| MCH^3^, pg | | 13.0-18.0 | 13.55 | 13.83 | 13.85 | 0.211 | 0.2280 | 0.0941 |
| MCHC^3^, g/dL | | 29.0-38.0 | 34.23 | 34.32 | 34.29 | 0.255 | 0.9493 | 0.7564 |
| Platelets, 10^3^/µL | | 300-700 | 353.27 | 293.70 | 350.46 | 70.371 | 0.6783 | 0.6787 |
| ^1^Diets enriched in resistant starch (HRS), a fiber-prebiotic-probiotic blend (FPPB), or a fiber-prebiotic-probiotic blend + immuno-modulating ingredients (iFPPB).  ^2^Reference ranges were provided from the University of Illinois Veterinary Diagnostic Laboratory.  ^3^MCH: mean corpuscular hemoglobin; MCHC: mean corpuscular hemoglobin concentration. | | | | | | | | |

| **Supplementary Table 6.** Bacterial species that were greater in feces of cats fed FPPB or iFPPB than those fed ERS | | | | |
| --- | --- | --- | --- | --- |
| Phylum | Genus | Species | Log 2 FC | Adjusted P Value |
| Actinobacteria | *Senegalimassilia* | *Senegalimassilia_anaerobia* | 4.1 | 4.0E-11 |
|  | *Actinomyces* | *Actinomyces_sp._oral_taxon_877* | 3.2 | 1.3E-03 |
|  | *Slackia* | *Slackia_piriformis* | 2.7 | 6.9E-10 |
|  | *Alloscardovia* | *Alloscardovia_sp._HMSC034E08* | 2.6 | 5.1E-03 |
| Bacteroidetes | *Alistipes* | *Alistipes_putredinis* | 8.2 | 2.5E-04 |
|  | *Bacteroides* | *Bacteroides_vulgatus* | 7.0 | 2.6E-10 |
|  |  | *Bacteroides_stercoris* | 6.7 | 1.1E-10 |
|  |  | *Bacteroides_sp._4_3_47FAA* | 6.7 | 1.2E-04 |
|  |  | *unspecified species* | 6.1 | 1.5E-09 |
|  |  | *Bacteroides_dorei* | 5.4 | 6.5E-04 |
|  |  | *Bacteroides_sp._HMSC068A09* | 4.8 | 1.7E-03 |
|  |  | *Bacteroides_ovatus* | 4.4 | 3.0E-03 |
|  |  | *Bacteroides_thetaiotaomicron* | 4.4 | 5.5E-03 |
|  | *Parabacteroides* | *Parabacteroides_merdae* | 6.5 | 1.9E-06 |
|  |  | *unspecified species* | 5.7 | 2.2E-04 |
|  |  | *Parabacteroides_distasonis* | 4.4 | 2.4E-03 |
|  | *Odoribacter* | *Odoribacter_laneus* | 4.1 | 1.1E-03 |
| Firmicutes | *Aerococcus* | *Aerococcus_urinaeequi* | 25.7 | 1.8E-16 |
|  |  | *unspecified species* | 23.1 | 1.7E-18 |
|  |  | *Aerococcus_viridans* | 8.0 | 3.4E-04 |
|  | *Acetivibrio* | *Acetivibrio_ethanolgignens* | 3.8 | 4.9E-11 |
|  | *Absiella* | *Absiella_dolichum* | 2.3 | 1.8E-03 |
|  | *Anaeromassilibacillus* | *Anaeromassilibacillus_sp._Marseille-P3371* | 3.9 | 2.4E-08 |
|  | *Anaerosporobacter* | *Anaerosporobacter_mobilis* | 2.3 | 1.5E-03 |
|  | *Agathobaculum* | *Agathobaculum_desmolans* | 2.2 | 1.5E-03 |
|  | *Anaerotruncus* | *Anaerotruncus_sp._G3(2012)* | 3.9 | 5.2E-06 |
|  |  | *Anaerotruncus_colihominis* | 3.0 | 1.2E-07 |
|  | *Angelakisella* | *Angelakisella_massiliensis* | 3.6 | 4.0E-11 |
|  | *Anaerotignum* | *Anaerotignum_lactatifermentans* | 2.6 | 6.9E-04 |
|  | *Anaerostipes* | *Anaerostipes_hadrus* | 2.3 | 5.8E-04 |
|  |  | *Anaerostipes_sp._3_2_56FAA* | 2.7 | 5.5E-06 |
|  | *Bacillus* | *Bacillus_coagulans* | 4.6 | 2.3E-08 |
|  |  | *Bacillus_smithii* | 2.6 | 7.4E-03 |
|  | *Bariatricus* | *Bariatricus_massiliensis* | 4.4 | 2.0E-04 |
|  | *Bittarella* | *Bittarella_massiliensis* | 3.9 | 5.6E-04 |
|  | *Blautia* | *[Ruminococcus]_torques* | 5.8 | 4.5E-08 |
|  |  | *Blautia_hydrogenotrophica* | 5.1 | 1.5E-08 |
|  |  | *unspecified species* | 4.1 | 9.2E-11 |
|  |  | *Blautia_wexlerae* | 4.1 | 6.1E-11 |
|  |  | *Blautia_sp._Marseille-P2398* | 4.1 | 2.5E-15 |
|  |  | *Blautia_obeum* | 3.9 | 1.1E-13 |
|  |  | *Blautia_schinkii* | 3.4 | 1.3E-12 |
|  |  | *Blautia_sp._Marseille-P3087* | 3.4 | 9.7E-13 |
|  |  | *Blautia_sp._Marseille-P3201T* | 3.2 | 9.2E-06 |
|  |  | *[Ruminococcus]_gnavus* | 2.9 | 1.4E-09 |
|  |  | *Blautia_producta* | 2.8 | 5.0E-10 |
|  |  | *Blautia_sp._SF-50* | 2.7 | 5.2E-06 |
|  |  | *Blautia_hansenii* | 2.6 | 2.0E-06 |
|  |  | *Blautia_sp._KLE_1732* | 2.5 | 1.0E-03 |
|  | *Butyricicoccus* | *Butyricicoccus_pullicaecorum* | 5.5 | 6.4E-12 |
|  | *Butyrivibrio* | *Butyrivibrio_crossotus* | 3.3 | 4.4E-03 |
|  | *Catonella* | *Catonella_morbi* | 5.0 | 2.6E-06 |
|  | *Clostridioides* | *Clostridioides_difficile* | 2.1 | 4.2E-04 |
|  | *Clostridium* | *Clostridium_sp._ATCC_BAA-442* | 4.0 | 2.4E-05 |
|  |  | *Clostridium_sp._ASF502* | 4.0 | 2.6E-07 |
|  |  | *Clostridium_sp._KLE_1755* | 3.8 | 2.3E-04 |
|  |  | *Clostridium_sp._M62/1* | 3.6 | 2.1E-06 |
|  |  | *Clostridium_sp._AT4* | 3.5 | 2.1E-13 |
|  |  | *Clostridium_sp._SS2/1* | 3.2 | 7.9E-06 |
|  |  | *Clostridium_sp._ATCC_29733* | 3.0 | 5.3E-03 |
|  |  | *Clostridium_cochlearium* | 3.0 | 1.1E-03 |
|  |  | *Clostridium_novyi* | 2.8 | 3.0E-03 |
|  |  | *Clostridium_sp._HMSC19A11* | 2.6 | 7.1E-08 |
|  |  | *Clostridium_polynesiense* | 2.6 | 8.3E-04 |
|  |  | *Clostridium_sp._Marseille-P3244* | 2.5 | 6.9E-03 |
|  |  | *Clostridium_sp._SN20* | 2.4 | 1.1E-03 |
|  |  | *Clostridium_phoceensis* | 2.2 | 1.1E-03 |
|  | *Coprobacillus* | *Coprobacillus_sp._D7* | 3.1 | 5.6E-07 |
|  |  | *Coprobacillus_sp._8_2_54BFAA* | 2.3 | 1.0E-04 |
|  |  | *Coprobacillus_sp._29_1* | 2.3 | 9.6E-05 |
|  |  | *Coprobacillus_sp._3_3_56FAA* | 2.1 | 2.4E-03 |
|  | *Coprococcus* | *Coprococcus_eutactus* | 2.5 | 1.3E-09 |
|  |  | *Coprococcus_sp._HPP0048* | 2.4 | 4.2E-09 |
|  | *Dorea* | *Dorea_sp._5-2* | 3.1 | 9.3E-07 |
|  |  | *Dorea_longicatena* | 2.1 | 3.8E-09 |
|  | *Eisenbergiella* | *Eisenbergiella_tayi* | 3.5 | 6.3E-06 |
|  | *Emergencia* | *Emergencia_timonensis* | 3.3 | 6.5E-07 |
|  | *Enterococcus* | *Enterococcus_sp._3G6_DIV0642* | 18.8 | 2.9E-08 |
|  |  | *Enterococcus_sp._HMSC069A01* | 5.3 | 1.6E-04 |
|  |  | *Enterococcus_sp._HMSC065H03* | 4.9 | 5.1E-03 |
|  |  | *Enterococcus_gilvus* | 3.9 | 7.0E-03 |
|  |  | *Enterococcus_sp._10F3_DIV0382* | 3.8 | 6.7E-03 |
|  |  | *Enterococcus_gallinarum* | 3.5 | 9.4E-03 |
|  | *Eubacterium* | *Eubacterium_plexicaudatum* | 4.5 | 1.9E-07 |
|  |  | *unspecified species* | 4.4 | 8.4E-07 |
|  |  | *Eubacterium_limosum* | 3.4 | 3.7E-09 |
|  |  | *Eubacterium_ventriosum* | 3.1 | 2.9E-07 |
|  |  | *Eubacterium_sp._YI* | 3.0 | 2.4E-03 |
|  |  | *Eubacterium_sp._3_1_31* | 3.0 | 1.8E-04 |
|  |  | *[Eubacterium]_hallii* | 2.8 | 6.1E-11 |
|  | *Faecalicatena* | *Faecalicatena_contorta* | 2.1 | 2.8E-04 |
|  | *Faecalicoccus* | *Faecalicoccus_pleomorphus* | 3.2 | 5.1E-10 |
|  | *Faecalitalea* | *Faecalitalea_cylindroides* | 4.3 | 4.2E-08 |
|  | *Flavonifractor* | *Flavonifractor_plautii* | 3.1 | 5.5E-08 |
|  | *Fournierella* | *Fournierella_massiliensis* | 4.2 | 9.2E-18 |
|  | *Fusicatenibacter* | *Fusicatenibacter_sp._2789STDY5834925* | 4.6 | 1.1E-07 |
|  | *Gemmiger* | *Gemmiger_formicilis* | 3.2 | 1.5E-09 |
|  | *Geobacillus* | *unspecified species* | 2.7 | 2.3E-03 |
|  | *Geobacillus* | *Geobacillus_sp._WCH70* | 2.6 | 4.9E-03 |
|  | *Hespellia* | *Hespellia_stercorisuis* | 2.9 | 4.3E-05 |
|  | *Holdemanella* | *Holdemanella_biformis* | 5.5 | 1.6E-19 |
|  | *Holdemania* | *Holdemania_sp._Marseille-P2844* | 2.9 | 8.1E-04 |
|  |  | *Holdemania_massiliensis* | 2.5 | 1.0E-05 |
|  |  | *Holdemania_filiformis* | 2.4 | 1.8E-03 |
|  | *Hungatella* | *Hungatella_hathewayi* | 2.5 | 2.6E-07 |
|  | *Ileibacterium* | *Ileibacterium_massiliense* | 2.4 | 7.8E-03 |
|  | *Intestinimonas* | *Intestinimonas_butyriciproducens* | 3.9 | 3.4E-05 |
|  |  | *Intestinimonas_massiliensis* | 3.4 | 4.2E-04 |
|  | *Lachnoclostridium* | *Lachnoclostridium_sp._YL32* | 4.6 | 2.4E-07 |
|  |  | *[Clostridium]_saccharolyticum* | 4.0 | 1.7E-09 |
|  |  | *[Clostridium]_lavalense* | 3.9 | 1.4E-04 |
|  |  | *unspecified species* | 3.5 | 2.2E-15 |
|  |  | *Lachnoclostridium_phocaeense* | 3.4 | 9.7E-08 |
|  |  | *[Clostridium]_glycyrrhizinilyticum* | 2.8 | 7.6E-08 |
|  |  | *[Clostridium]_bolteae* | 2.8 | 1.0E-10 |
|  |  | *[Clostridium]_clostridioforme* | 2.7 | 4.2E-12 |
|  |  | *[Clostridium]_symbiosum* | 2.7 | 9.6E-05 |
|  |  | *[Clostridium]_scindens* | 2.0 | 1.1E-04 |
|  | *Lachnospira* | *Lachnospira_pectinoschiza* | 3.8 | 3.3E-04 |
|  | *Lactobacillus* | *Lactobacillus_animalis* | 8.5 | 1.1E-11 |
|  |  | *Lactobacillus_murinus* | 8.3 | 6.5E-10 |
|  |  | *unspecified species* | 5.8 | 1.1E-09 |
|  |  | *Lactobacillus_rogosae* | 3.5 | 4.7E-04 |
|  |  | *Lactobacillus_aviarius* | 2.9 | 2.6E-03 |
|  | *Leuconostoc* | *Leuconostoc_citreum* | 5.3 | 7.4E-03 |
|  | *Mageeibacillus* | *Mageeibacillus_indolicus* | 3.4 | 7.8E-03 |
|  | *Massilioclostridium* | *Massilioclostridium_coli* | 3.0 | 1.1E-03 |
|  | *Merdimonas* | *Merdimonas_faecis* | 3.5 | 7.8E-11 |
|  | *Mordavella* | *Mordavella_sp._Marseille-P3756* | 3.1 | 3.5E-08 |
|  | *Murdochiella* | *Murdochiella_vaginalis* | 3.1 | 2.4E-03 |
|  | *Negativibacillus* | *Negativibacillus_massiliensis* | 4.0 | 1.8E-12 |
|  | *Neglecta* | *Neglecta_timonensis* | 2.5 | 1.2E-03 |
|  | *Oscillibacter* | *unspecified species* | 6.0 | 1.0E-07 |
|  | *Pediococcus* | *Pediococcus_acidilactici* | 7.4 | 8.7E-09 |
|  |  | *Pediococcus_inopinatus* | 3.5 | 6.3E-04 |
|  |  | *Pediococcus_damnosus* | 3.2 | 2.7E-04 |
|  | *Phocea* | *Phocea_massiliensis* | 4.9 | 6.8E-05 |
|  | *Provencibacterium* | *Provencibacterium_massiliense* | 2.8 | 2.3E-07 |
|  | *Pseudoflavonifractor* | *Pseudoflavonifractor_capillosus* | 3.8 | 1.8E-12 |
|  |  | *Pseudoflavonifractor_sp._Marseille-P3106* | 3.3 | 4.5E-04 |
|  | *Roseburia* | *Roseburia_hominis* | 4.2 | 4.2E-11 |
|  |  | *Roseburia_inulinivorans* | 2.9 | 6.3E-12 |
|  | *Ruminiclostridium* | *[Clostridium]_leptum* | 4.1 | 1.6E-04 |
|  | *Ruminococcus* | *Ruminococcus_faecis* | 6.7 | 3.5E-07 |
|  |  | *Ruminococcus_bicirculans* | 3.6 | 1.1E-04 |
|  |  | *Ruminococcus_sp._AT10* | 2.8 | 5.9E-15 |
|  |  | *Ruminococcus_lactaris* | 2.3 | 4.3E-07 |
|  | *Ruthenibacterium* | *Ruthenibacterium_lactatiformans* | 2.9 | 1.4E-04 |
|  | *Sharpea* | *Sharpea_azabuensis* | 2.4 | 4.9E-03 |
|  | *Solobacterium* | *Solobacterium_moorei* | 4.6 | 5.1E-14 |
|  | *Streptococcus* | *Streptococcus_canis* | 6.1 | 1.6E-03 |
|  |  | *Streptococcus_entericus* | 5.2 | 1.1E-03 |
|  |  | *Streptococcus_agalactiae* | 5.0 | 3.4E-07 |
|  |  | *Streptococcus_parauberis* | 4.1 | 6.1E-08 |
|  |  | *Streptococcus_parasanguinis* | 3.7 | 2.8E-07 |
|  |  | *Streptococcus_mitis* | 3.6 | 9.3E-03 |
|  |  | *Streptococcus_intermedius* | 3.4 | 9.3E-03 |
|  | *Subdoligranulum* | *Subdoligranulum_sp._4_3_54A2FAA* | 3.5 | 1.5E-05 |
|  |  | *Subdoligranulum_variabile* | 3.2 | 6.4E-16 |
|  | *Traorella* | *Traorella_massiliensis* | 3.2 | 1.2E-04 |
| Proteobacteria | *Escherichia* | *unspecified species* | 3.8 | 3.5E-03 |
| Tenericutes | *Mycoplasma* | *Mycoplasma_hominis* | 3.9 | 4.9E-03 |

| **Supplementary Table 7.** Bacterial species that were lower in feces of cats fed FPPB or iFPPB than those fed ERS | | | | |
| --- | --- | --- | --- | --- |
| Phylum | Genus | Species | Log 2 FC | Adjusted P Value |
| Actinobacteria | *Actinomyces* | *Actinomyces_europaeus* | -3.3 | 1.4E-07 |
|  | *Atopobium* | *Atopobium_minutum* | -2.2 | 8.3E-04 |
|  | *Bifidobacterium* | *Bifidobacterium_pseudolongum* | -2.0 | 7.3E-04 |
|  |  | *Bifidobacterium_callitrichos* | -2.5 | 2.6E-07 |
|  |  | *Bifidobacterium_stellenboschense* | -2.5 | 4.1E-06 |
|  |  | *Bifidobacterium_choerinum* | -2.8 | 3.0E-13 |
|  |  | *Bifidobacterium_sp._TRE_H* | -3.1 | 1.7E-05 |
|  |  | *Bifidobacterium_saguini* | -3.2 | 3.0E-08 |
|  |  | *Bifidobacterium_sp._12_1_47BFAA* | -3.3 | 2.7E-08 |
|  |  | *Bifidobacterium_scardovii* | -3.4 | 8.8E-09 |
|  |  | *Bifidobacterium_boum* | -3.4 | 3.6E-07 |
|  |  | *Bifidobacterium_catenulatum* | -3.5 | 4.5E-09 |
|  |  | *Bifidobacterium_longum* | -3.5 | 4.1E-09 |
|  |  | *Bifidobacterium_sp._TRE_D* | -3.6 | 1.5E-08 |
|  |  | *Bifidobacterium_aesculapii* | -3.6 | 4.2E-12 |
|  |  | *Bifidobacterium_dentium* | -3.6 | 9.4E-12 |
|  |  | *Bifidobacterium_sp._AGR2158* | -3.7 | 1.8E-05 |
|  |  | *Bifidobacterium_sp._TRE_1* | -3.7 | 7.2E-10 |
|  |  | *Bifidobacterium_reuteri* | -3.7 | 5.0E-08 |
|  |  | *Bifidobacterium_bifidum* | -3.8 | 9.2E-15 |
|  |  | *Bifidobacterium_angulatum* | -3.8 | 2.8E-12 |
|  |  | *Bifidobacterium_breve* | -3.9 | 1.5E-11 |
|  |  | *Bifidobacterium_kashiwanohense* | -3.9 | 7.1E-12 |
|  |  | *Bifidobacterium_cuniculi* | -4.6 | 4.0E-24 |
|  |  | *Bifidobacterium_adolescentis* | -4.7 | 2.9E-14 |
|  |  | *Bifidobacterium_thermophilum* | -5.9 | 2.4E-23 |
|  | *Corynebacterium* | *Corynebacterium_pyruviciproducens* | -2.1 | 1.4E-06 |
|  | *Olsenella* | *Olsenella_profusa* | -2.1 | 4.8E-05 |
|  |  | *Olsenella_sp._KH1P3* | -2.5 | 8.6E-03 |
|  |  | *Olsenella_sp._kh2p3* | -2.9 | 1.5E-05 |
|  |  | *Olsenella_scatoligenes* | -2.9 | 1.5E-04 |
|  |  | *Olsenella_sp._KH3B4* | -3.0 | 5.1E-06 |
|  |  | *Olsenella_sp._Marseille-P2300* | -3.3 | 5.4E-06 |
|  |  | *Olsenella_umbonata* | -3.8 | 1.3E-07 |
|  | *Rhodococcus* | *unspecified species* | -2.9 | 5.1E-06 |
| Firmicutes | *Acidaminococcus* | *Acidaminococcus_intestini* | -4.6 | 2.2E-06 |
|  | *Anaerocolumna* | *Anaerocolumna_jejuensis* | -6.9 | 2.1E-19 |
|  | *Clostridium* | *Clostridium_sp._7_3_54FAA* | -3.3 | 4.7E-12 |
|  |  | *Clostridium_puniceum* | -4.3 | 7.2E-04 |
|  | *Eubacterium* | *Eubacterium_pyruvativorans* | -5.3 | 1.2E-04 |
|  | *Fontibacillus* | *Fontibacillus_panacisegetis* | -4.9 | 4.3E-09 |
|  | *Lachnoclostridium* | *[Clostridium]_aerotolerans* | -5.9 | 9.8E-13 |
|  | *Lactobacillus* | *Lactobacillus_agilis* | -3.1 | 8.2E-03 |
|  |  | *Lactobacillus_acetotolerans* | -4.1 | 3.2E-03 |
|  |  | *Lactobacillus_ruminis* | -5.0 | 6.9E-07 |
|  | *Levyella* | *Levyella_massiliensis* | -2.5 | 1.9E-04 |
|  | *Mitsuokella* | *Mitsuokella_jalaludinii* | -4.0 | 5.3E-03 |
|  |  | *Mitsuokella_multacida* | -6.3 | 3.1E-06 |
|  | *Mobilibacterium* | *Mobilibacterium_timonense* | -5.6 | 1.4E-04 |
|  | *Mogibacterium* | *Mogibacterium_timidum* | -8.1 | 5.4E-04 |
|  | *Paenibacillus* | *unspecified species* | -4.4 | 2.1E-13 |
|  |  | *Paenibacillus_xylanexedens* | -4.5 | 4.3E-06 |
|  | *Pelosinus* | *Pelosinus_fermentans* | -5.1 | 1.6E-11 |
|  | *Selenomonas* | *Selenomonas_bovis* | -4.1 | 3.8E-04 |
| Proteobacteria | *Campylobacter* | *Campylobacter_upsaliensis* | -4.0 | 5.4E-04 |
|  |  | *Campylobacter_helveticus* | -4.2 | 3.7E-04 |
|  | *Helicobacter* | *Helicobacter_winghamensis* | -4.3 | 2.5E-03 |
|  | *Pseudomonas* | *Pseudomonas_putida* | -2.5 | 4.9E-03 |

| **Supplementary Table 8.** Kyoto Encyclopedia of Genes and Genomes Orthology (KO) terms that were greater in cats fed ERS than those fed FPPB or iFPPB^1^ | | | |
| --- | --- | --- | --- |
| KO terms | Description | Log 2 FC* | Adjusted P Value** |
| K01200 | pullulanase | -2.92 | 1.29E-07 |
| K01218 | mannan endo-1,4-beta-mannosidase | -2.81 | 3.19E-06 |
| K18567 | MFS transporter, DHA1 family, purine base/nucleoside efflux pump | -2.78 | 1.67E-06 |
| K01858 | myo-inositol-1-phosphate synthase | -2.73 | 6.61E-08 |
| K07442 | tRNA (adenine57-N1/adenine58-N1)-methyltransferase catalytic subunit | -2.72 | 2.65E-08 |
| K12583 | phosphatidylinositol alpha 1,6-mannosyltransferase | -2.68 | 6.13E-07 |
| K17829 | crotonyl-CoA reductase | -2.66 | 1.98E-06 |
| K01182 | oligo-1,6-glucosidase | -2.64 | 5.09E-07 |
| K07692 | two-component system, NarL family, response regulator DegU | -2.64 | 2.98E-07 |
| K01057 | 6-phosphogluconolactonase | -2.60 | 3.70E-06 |
| K03453 | bile acid:Na+ symporter, BASS family | -2.59 | 1.22E-08 |
| K00240 | succinate dehydrogenase / fumarate reductase, iron-sulfur subunit | -2.58 | 3.31E-08 |
| K03929 | para-nitrobenzyl esterase | -2.57 | 3.62E-07 |
| K11263 | acetyl-CoA/propionyl-CoA carboxylase, biotin carboxylase, biotin carboxyl carrier protein | -2.56 | 4.45E-07 |
| K16649 | rhamnopyranosyl-N-acetylglucosaminyl-diphospho-decaprenol beta-1,3/1,4-galactofuranosyltransferase | -2.55 | 3.71E-07 |
| K03707 | thiaminase | -2.54 | 6.50E-06 |
| K18968 | diguanylate cyclase | -2.54 | 3.46E-07 |
| K02205 | arginine/ornithine permease | -2.54 | 2.22E-06 |
| K05794 | tellurite resistance protein TerC | -2.51 | 1.50E-06 |
| K18205 | non-reducing end beta-L-arabinofuranosidase | -2.49 | 1.81E-09 |
| K07230 | periplasmic iron binding protein | -2.45 | 1.77E-06 |
| K03338 | 5-dehydro-2-deoxygluconokinase | -2.38 | 8.90E-07 |
| K13571 | proteasome accessory factor A | -2.37 | 1.67E-06 |
| K00936 | two-component system, sensor histidine kinase PdtaS | -2.37 | 4.01E-07 |
| K01760 | cysteine-S-conjugate beta-lyase | -2.37 | 3.77E-08 |
| K07259 | serine-type D-Ala-D-Ala carboxypeptidase/endopeptidase (penicillin-binding protein 4) | -2.36 | 3.73E-07 |
| K15531 | oligosaccharide reducing-end xylanase | -2.34 | 1.12E-06 |
| K13288 | oligoribonuclease | -2.34 | 1.07E-07 |
| K16856 | ureidoglycolate lyase | -2.34 | 1.22E-08 |
| K00324 | H+-translocating NAD(P) transhydrogenase subunit alpha | -2.33 | 2.70E-08 |
| K14215 | trans,polycis-decaprenyl diphosphate synthase | -2.33 | 2.19E-06 |
| K00702 | cellobiose phosphorylase | -2.33 | 4.05E-07 |
| K03658 | DNA helicase IV | -2.32 | 8.22E-07 |
| K07183 | two-component system, response regulator / RNA-binding antiterminator | -2.32 | 8.41E-07 |
| K20814 | Pup amidohydrolase | -2.32 | 1.38E-06 |
| K06221 | 2,5-diketo-D-gluconate reductase A | -2.32 | 8.80E-11 |
| K16147 | starch synthase (maltosyl-transferring) | -2.32 | 2.58E-06 |
| K07503 | endonuclease | -2.31 | 3.79E-06 |
| K01997 | branched-chain amino acid transport system permease protein | -2.30 | 1.42E-10 |
| K07407 | alpha-galactosidase | -2.30 | 1.80E-05 |
| K01897 | long-chain acyl-CoA synthetase | -2.30 | 4.30E-08 |
| K18907 | GntR family transcriptional regulator, regulator for abcA and norABC | -2.30 | 4.32E-08 |
| K10805 | acyl-CoA thioesterase II | -2.27 | 2.17E-08 |
| K01704 | 3-isopropylmalate/(R)-2-methylmalate dehydratase small subunit | -2.27 | 9.33E-08 |
| K07777 | two-component system, NarL family, sensor histidine kinase DegS | -2.25 | 4.26E-06 |
| K18473 | acetoacetyl-[acyl-carrier protein] synthase | -2.25 | 5.28E-06 |
| K03772 | FKBP-type peptidyl-prolyl cis-trans isomerase FkpA | -2.25 | 2.32E-07 |
| K01894 | glutamyl-Q tRNA(Asp) synthetase | -2.24 | 7.69E-08 |
| K00657 | diamine N-acetyltransferase | -2.24 | 1.99E-08 |
| K13051 | L-asparaginase / beta-aspartyl-peptidase | -2.24 | 9.93E-08 |
| K15524 | mannosylglycerate hydrolase | -2.24 | 7.45E-08 |
| K00674 | 2,3,4,5-tetrahydropyridine-2,6-dicarboxylate N-succinyltransferase | -2.23 | 4.36E-08 |
| K00705 | 4-alpha-glucanotransferase | -2.23 | 3.82E-08 |
| K05364 | penicillin-binding protein A | -2.23 | 4.66E-07 |
| K00018 | glycerate dehydrogenase | -2.23 | 8.11E-07 |
| K01703 | 3-isopropylmalate/(R)-2-methylmalate dehydratase large subunit | -2.22 | 7.23E-08 |
| K00886 | polyphosphate glucokinase | -2.21 | 5.90E-06 |
| K07053 | 3',5'-nucleoside bisphosphate phosphatase | -2.21 | 4.19E-08 |
| K19285 | FMN reductase (NADPH) | -2.21 | 1.93E-05 |
| K08301 | ribonuclease G | -2.21 | 5.42E-09 |
| K11734 | aromatic amino acid transport protein AroP | -2.20 | 5.73E-08 |
| K17235 | arabinooligosaccharide transport system permease protein | -2.20 | 5.86E-07 |
| K13940 | dihydroneopterin aldolase / 2-amino-4-hydroxy-6-hydroxymethyldihydropteridine diphosphokinase | -2.18 | 2.73E-06 |
| K08289 | phosphoribosylglycinamide formyltransferase 2 | -2.18 | 1.14E-08 |
| K03817 | ribosomal-protein-serine acetyltransferase | -2.17 | 9.46E-09 |
| K02006 | cobalt/nickel transport system ATP-binding protein | -2.17 | 1.47E-06 |
| K03307 | solute:Na+ symporter, SSS family | -2.17 | 9.81E-10 |
| K02523 | octaprenyl-diphosphate synthase | -2.16 | 2.51E-09 |
| K00254 | dihydroorotate dehydrogenase | -2.16 | 2.68E-08 |
| K07768 | two-component system, OmpR family, sensor histidine kinase SenX3 | -2.16 | 2.47E-07 |
| K01673 | carbonic anhydrase | -2.16 | 8.22E-07 |
| K16918 | acetoin utilization transport system permease protein | -2.16 | 5.83E-07 |
| K01996 | branched-chain amino acid transport system ATP-binding protein | -2.15 | 4.63E-11 |
| K00990 | uridylyltransferase | -2.14 | 5.82E-08 |
| K13243 | c-di-GMP-specific phosphodiesterase | -2.14 | 4.58E-05 |
| K04518 | prephenate dehydratase | -2.14 | 4.38E-06 |
| K13892 | glutathione transport system ATP-binding protein | -2.13 | 5.67E-10 |
| K15584 | nickel transport system substrate-binding protein | -2.13 | 9.86E-08 |
| K02483 | two-component system, OmpR family, response regulator | -2.13 | 3.36E-10 |
| K00851 | gluconokinase | -2.12 | 7.19E-07 |
| K20866 | glucose-1-phosphatase | -2.12 | 9.89E-10 |
| K17318 | putative aldouronate transport system substrate-binding protein | -2.12 | 3.37E-06 |
| K09016 | putative pyrimidine permease RutG | -2.12 | 1.92E-08 |
| K07757 | sugar-phosphatase | -2.11 | 1.75E-07 |
| K03575 | A/G-specific adenine glycosylase | -2.11 | 1.30E-08 |
| K07778 | two-component system, NarL family, sensor histidine kinase DesK | -2.11 | 1.78E-05 |
| K04772 | serine protease DegQ | -2.10 | 9.55E-08 |
| K01664 | para-aminobenzoate synthetase component II | -2.09 | 1.17E-08 |
| K01621 | xylulose-5-phosphate/fructose-6-phosphate phosphoketolase | -2.08 | 8.90E-06 |
| K16148 | alpha-maltose-1-phosphate synthase | -2.06 | 8.90E-06 |
| K01681 | aconitate hydratase | -2.05 | 2.08E-08 |
| K01207 | beta-N-acetylhexosaminidase | -2.05 | 3.00E-09 |
| K01761 | methionine-gamma-lyase | -2.05 | 9.27E-07 |
| K00766 | anthranilate phosphoribosyltransferase | -2.04 | 2.93E-11 |
| K00982 | [glutamine synthetase] adenylyltransferase / [glutamine synthetase]-adenylyl-L-tyrosine phosphorylase | -2.04 | 7.83E-08 |
| K08884 | serine/threonine protein kinase, bacterial | -2.04 | 1.45E-06 |
| K01077 | alkaline phosphatase | -2.03 | 3.16E-08 |
| K09810 | lipoprotein-releasing system ATP-binding protein | -2.03 | 3.10E-10 |
| K05349 | beta-glucosidase | -2.02 | 3.64E-07 |
| K01854 | UDP-galactopyranose mutase | -2.02 | 1.80E-08 |
| K06155 | Gnt-I system high-affinity gluconate transporter | -2.02 | 3.79E-06 |
| K07282 | gamma-polyglutamate biosynthesis protein CapA | -2.02 | 2.20E-06 |
| K01494 | dCTP deaminase | -2.02 | 1.73E-08 |
| K07243 | high-affinity iron transporter | -2.02 | 3.33E-06 |
| K03587 | cell division protein FtsI (penicillin-binding protein 3) | -2.02 | 8.72E-09 |
| K03727 | ATP-dependent RNA helicase HelY | -2.01 | 1.03E-06 |
| K01739 | cystathionine gamma-synthase | -2.01 | 9.51E-07 |
| K01696 | tryptophan synthase beta chain | -2.00 | 4.92E-09 |
| K01903 | succinyl-CoA synthetase beta subunit | -2.00 | 2.71E-06 |
| ^1^Diets enriched in resistant starch (ERS), a fiber-prebiotic-probiotic blend (FPPB), or a fiber-prebiotic-probiotic blend + immuno-modulating ingredients (iFPPB).  *FC: fold change.  **P values were adjusted using the false discovery rate. | | | |

| **Supplementary Table 9.** Kyoto Encyclopedia of Genes and Genomes Orthology (KO) terms that were greater in cats fed FPPB or iFPPB than those fed ERS^1^ | | | |
| --- | --- | --- | --- |
| KO terms | Description | Log 2 FC* | Adjusted P Value** |
| K03529 | chromosome segregation protein | 2.01 | 2.95E-07 |
| K12573 | ribonuclease R | 2.04 | 1.06E-05 |
| K07568 | S-adenosylmethionine:tRNA ribosyltransferase-isomerase | 2.04 | 4.16E-06 |
| K03427 | type I restriction enzyme M protein | 2.04 | 2.98E-08 |
| K03572 | DNA mismatch repair protein MutL | 2.08 | 3.11E-03 |
| K03722 | ATP-dependent DNA helicase DinG | 2.08 | 9.43E-07 |
| K02111 | F-type H+/Na+-transporting ATPase subunit alpha | 2.08 | 5.72E-08 |
| K07668 | two-component system, OmpR family, response regulator VicR | 2.09 | 8.42E-09 |
| K11749 | regulator of sigma E protease | 2.10 | 6.82E-06 |
| K02837 | peptide chain release factor 3 | 2.11 | 3.43E-06 |
| K11085 | ATP-binding cassette, subfamily B, bacterial MsbA | 2.12 | 1.35E-06 |
| K02622 | topoisomerase IV subunit B | 2.12 | 4.69E-04 |
| K03495 | tRNA uridine 5-carboxymethylaminomethyl modification enzyme | 2.12 | 5.59E-04 |
| K00963 | UTP--glucose-1-phosphate uridylyltransferase | 2.12 | 3.11E-05 |
| K01223 | 6-phospho-beta-glucosidase | 2.12 | 9.82E-07 |
| K04083 | molecular chaperone Hsp33 | 2.13 | 1.98E-04 |
| K03151 | tRNA uracil 4-sulfurtransferase | 2.13 | 2.43E-06 |
| K01534 | Zn2+/Cd2+-exporting ATPase | 2.16 | 1.65E-04 |
| K02601 | transcription termination/antitermination protein NusG | 2.19 | 9.81E-09 |
| K01893 | asparaginyl-tRNA synthetase | 2.22 | 4.02E-08 |
| K11069 | spermidine/putrescine transport system substrate-binding protein | 2.23 | 1.28E-04 |
| K00864 | glycerol kinase | 2.23 | 1.11E-05 |
| K02112 | F-type H+/Na+-transporting ATPase subunit beta | 2.24 | 2.35E-07 |
| K03569 | rod shape-determining protein MreB and related proteins | 2.25 | 1.49E-06 |
| K03555 | DNA mismatch repair protein MutS | 2.25 | 4.82E-04 |
| K03546 | DNA repair protein SbcC/Rad50 | 2.25 | 1.87E-03 |
| K16786 | energy-coupling factor transport system ATP-binding protein | 2.26 | 2.78E-04 |
| K07010 | putative glutamine amidotransferase | 2.26 | 1.82E-06 |
| K09458 | 3-oxoacyl-[acyl-carrier-protein] synthase II | 2.27 | 3.58E-05 |
| K06131 | cardiolipin synthase A/B | 2.28 | 7.12E-04 |
| K01443 | N-acetylglucosamine-6-phosphate deacetylase | 2.29 | 1.02E-04 |
| K02621 | topoisomerase IV subunit A | 2.29 | 4.81E-03 |
| K01963 | acetyl-CoA carboxylase carboxyl transferase subunit beta | 2.32 | 2.35E-05 |
| K00847 | fructokinase | 2.33 | 3.41E-07 |
| K03446 | MFS transporter, DHA2 family, multidrug resistance protein | 2.33 | 6.61E-05 |
| K18908 | multidrug efflux pump | 2.34 | 1.17E-08 |
| K05565 | multicomponent Na+:H+ antiporter subunit A | 2.34 | 1.94E-07 |
| K07462 | single-stranded-DNA-specific exonuclease | 2.36 | 3.79E-09 |
| K03621 | phosphate acyltransferase | 2.38 | 1.31E-07 |
| K20141 | 2-oxoglutarate carboxylase small subunit | 2.38 | 1.01E-05 |
| K01809 | mannose-6-phosphate isomerase | 2.41 | 1.74E-06 |
| K02819 | trehalose PTS system EIIBC or EIIBCA component | 2.43 | 1.45E-06 |
| K19077 | two-component system, OmpR family, sensor histidine kinase GraS | 2.43 | 3.91E-07 |
| K01232 | maltose-6'-phosphate glucosidase | 2.47 | 8.73E-09 |
| K02794 | mannose PTS system EIIAB component | 2.48 | 1.83E-12 |
| K00645 | S-malonyltransferase | 2.48 | 1.04E-05 |
| K02082 | D-galactosamine 6-phosphate deaminase/isomerase | 2.48 | 9.91E-06 |
| K03169 | DNA topoisomerase III | 2.50 | 8.69E-13 |
| K01990 | ABC-2 type transport system ATP-binding protein | 2.50 | 1.53E-12 |
| K13292 | phosphatidylglycerol---prolipoprotein diacylglyceryl transferase | 2.51 | 1.45E-08 |
| K01478 | arginine deiminase | 2.57 | 6.42E-08 |
| K02824 | uracil permease | 2.59 | 1.42E-04 |
| K03581 | exodeoxyribonuclease V alpha subunit | 2.60 | 2.33E-05 |
| K00566 | tRNA-uridine 2-sulfurtransferase | 2.61 | 1.00E-07 |
| K16013 | ATP-binding cassette, subfamily C, bacterial CydD | 2.62 | 3.06E-03 |
| K07636 | two-component system, OmpR family, phosphate regulon sensor histidine kinase PhoR | 2.65 | 5.42E-12 |
| K00648 | 3-oxoacyl-[acyl-carrier-protein] synthase III | 2.65 | 6.35E-06 |
| K06213 | magnesium transporter | 2.66 | 4.33E-09 |
| K03294 | basic amino acid/polyamine antiporter, APA family | 2.70 | 1.85E-05 |
| K03491 | lichenan operon transcriptional antiterminator | 2.71 | 1.12E-07 |
| K19789 | DNA repair protein RadD | 2.72 | 1.52E-06 |
| K01738 | cysteine synthase | 2.74 | 1.92E-08 |
| K03311 | branched-chain amino acid:cation transporter | 2.80 | 3.34E-08 |
| K02099 | AraC family transcriptional regulator, arabinose operon regulatory protein | 2.87 | 1.09E-05 |
| K05846 | osmoprotectant transport system permease protein | 2.88 | 5.28E-06 |
| K09693 | teichoic acid transport system ATP-binding protein | 2.94 | 1.80E-08 |
| K09698 | nondiscriminating glutamyl-tRNA synthetase | 2.96 | 4.71E-08 |
| K04041 | fructose-1,6-bisphosphatase III | 2.97 | 1.42E-10 |
| K20118 | glucose PTS system EIICBA or EIICB component | 2.97 | 1.83E-12 |
| K19005 | lipoteichoic acid synthase | 2.98 | 1.14E-08 |
| K02761 | cellobiose PTS system EIIC component | 3.01 | 3.37E-11 |
| K03710 | GntR family transcriptional regulator | 3.06 | 4.55E-10 |
| K07652 | two-component system, OmpR family, sensor histidine kinase VicK | 3.08 | 3.94E-09 |
| K05823 | N-acetyldiaminopimelate deacetylase | 3.14 | 5.36E-07 |
| K05847 | osmoprotectant transport system ATP-binding protein | 3.15 | 3.16E-07 |
| K00625 | phosphate acetyltransferase | 3.17 | 6.01E-10 |
| K12555 | penicillin-binding protein 2A | 3.17 | 4.48E-10 |
| K04086 | ATP-dependent Clp protease ATP-binding subunit ClpL | 3.20 | 7.36E-05 |
| K04094 | methylenetetrahydrofolate--tRNA-(uracil-5-)-methyltransferase | 3.22 | 6.87E-10 |
| K18682 | ribonucrease Y | 3.28 | 9.55E-12 |
| K16323 | purine nucleoside transport protein | 3.33 | 2.59E-04 |
| K03697 | ATP-dependent Clp protease ATP-binding subunit ClpE | 3.34 | 4.69E-08 |
| K16898 | ATP-dependent helicase/nuclease subunit A | 3.37 | 4.40E-12 |
| K15986 | manganese-dependent inorganic pyrophosphatase | 3.40 | 3.43E-13 |
| K11144 | primosomal protein DnaI | 3.55 | 1.66E-13 |
| K08724 | penicillin-binding protein 2B | 3.66 | 8.29E-06 |
| K06286 | septation ring formation regulator | 3.66 | 1.22E-06 |
| K19220 | peptidoglycan DL-endopeptidase CwlS | 3.69 | 1.02E-11 |
| K11632 | bacitracin transport system permease protein | 3.77 | 1.57E-13 |
| K16899 | ATP-dependent helicase/nuclease subunit B | 3.79 | 1.83E-09 |
| K17473 | sigma-54 dependent transcriptional regulator, dga operon transcriptional activator | 3.80 | 7.00E-08 |
| K07456 | DNA mismatch repair protein MutS2 | 3.82 | 1.24E-09 |
| K09697 | sodium transport system ATP-binding protein | 3.82 | 4.14E-14 |
| K00687 | penicillin-binding protein 2B | 3.92 | 5.82E-07 |
| K03328 | polysaccharide transporter, PST family | 3.97 | 1.84E-08 |
| K18940 | two-component system, OmpR family, sensor histidine kinase ArlS | 4.04 | 1.69E-09 |
| K01533 | P-type Cu2+ transporter | 4.88 | 1.41E-04 |
| K03315 | Na+:H+ antiporter, NhaC family | 5.16 | 3.81E-20 |
| K00756 | pyrimidine-nucleoside phosphorylase | 5.48 | 1.44E-16 |
| K01361 | lactocepin | 5.62 | 1.60E-09 |
| K09952 | CRISPR-associated endonuclease Csn1 | 8.09 | 3.75E-15 |
| K00689 | dextransucrase | 9.03 | 6.01E-10 |
| K12953 | cation-transporting P-type ATPase F | 11.63 | 2.61E-15 |
| ^1^Diets enriched in resistant starch (ERS), a fiber-prebiotic-probiotic blend (FPPB), or a fiber-prebiotic-probiotic blend + immuno-modulating ingredients (iFPPB).  *FC: fold change.  **P values were adjusted using the false discovery rate. | | | |
