# Supplementary material for: Dietary enrichment of resistant starches or fibers differentially alter the feline fecal microbiome and metabolite profile
Source: Anim Microbiome. 2022 Dec 5;4:61. doi: 10.1186/s42523-022-00213-9 (PMC9720964; doi:10.1186/s42523-022-00213-9)
Supplement: Supplementary file 2 — Additional file 2: Fig. S1. Quality control measures of the microbiome data. (A) Sequencing coverage of bacterial taxa and samples in the dataset before quality control. Each column is a taxa of a sample. (B) Sequencing coverage of bacterial taxa and samples after removing rare taxa present in less than 5% of the samples. Each column is a taxa or a sample. (C) Abundance (x-axis) and prevalence (y-axis) of bacterial taxa in the dataset after quality control. Each dot is a taxa and they are faceted by phylum. Fig. S2. Rarefaction curves of fecal samples collected from cats fed the experimental diets. The curves show alpha diversity differences between diet groups. (A, B) Differences in Species Richness and Shannon’s diversity index (Shannon’s H) between the three diet groups. (C, D) No difference in these measures were observed between the periods during which the different diets were administered. Fig. S3. Fecal microbial composition of cats fed the experimental diets. (A) Screeplot showing the eigenvalues obtained from PCoA using Bray-Curtis (left) and Jensen-Shannon distances (right). (B-D) Principal Coordinate Analysis (PCoA) of species-level gut microbiomes using Jensen-Shannon distances. PERMANOVA was used to assess the association between diet and period with the gut microbiome composition. Kruskal-Wall is test was used to assess association between diet and the top two axes (PCo1, PCo2) followed by Dunn’s post-hoc test to evaluate the difference between the dietary groups. (B) A PCoA plot using Jensen-Shannon distance revealed significant association between diet groups (P = 0.0001, PERMANOVA). Period was also significant in this case but with much weaker significance (P = 0.034, PERMANOVA). (C) No difference in PCo1 or PCo2 between the periods (P = 0.12 and 0.41, respectively, Kruskal–Wallis test). (D) Diet was associated with both PCo1 and PCo2 (P < 0.0001 and 0.03, respectively, Kruskal Wallis test). Fig. S4. Volcano plots of fecal microbiota data from cats [file 42523_2022_213_MOESM2_ESM.docx]

**
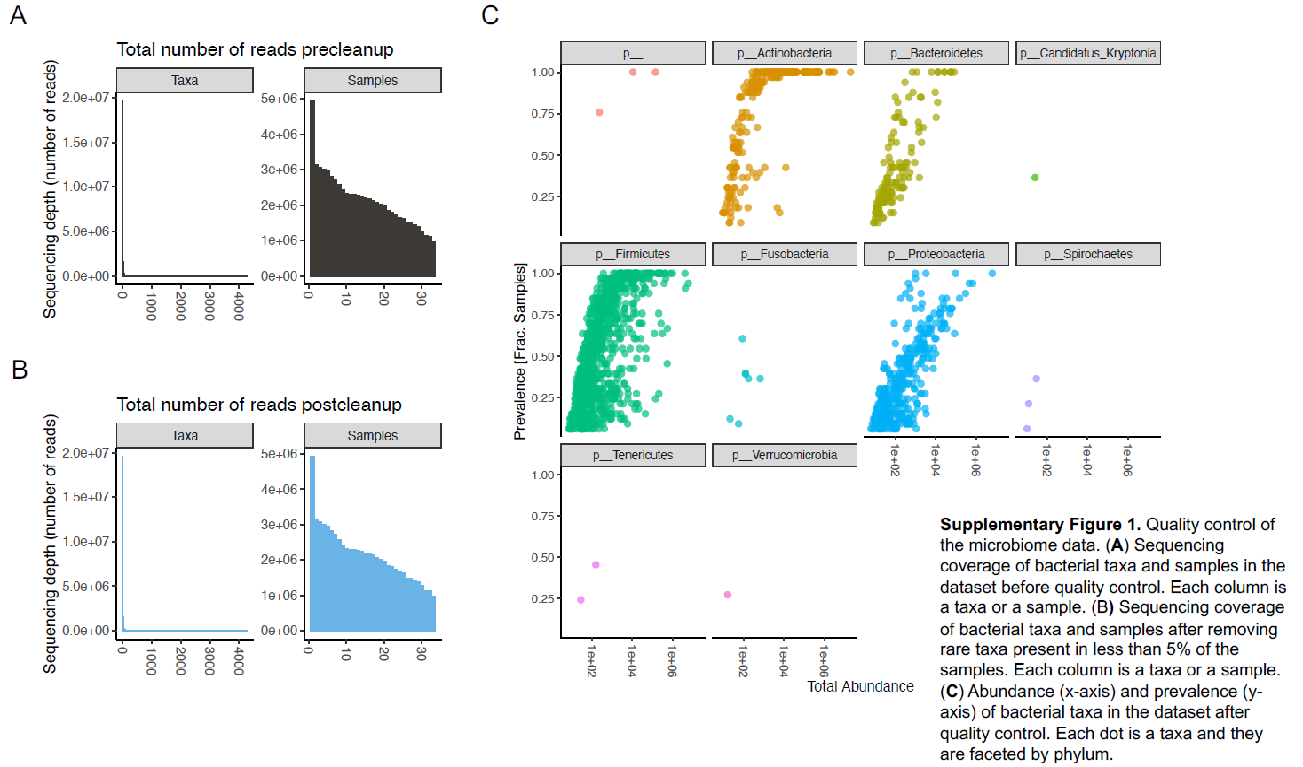
**

**Supplementary Figure 1.** Quality control measures of the microbiome data**. (A)** Sequencing coverage of bacterial taxa and samples in the dataset before quality control. Each column is a taxa of a sample. **(B)** Sequencing coverage of bacterial taxa and samples after removing rare taxa present in less than 5% of the samples. Each column is a taxa or a sample. **(C)** Abundance (x-axis) and prevalence (y-axis) of bacterial taxa in the dataset after quality control. Each dot is a taxa and they are faceted by phylum.


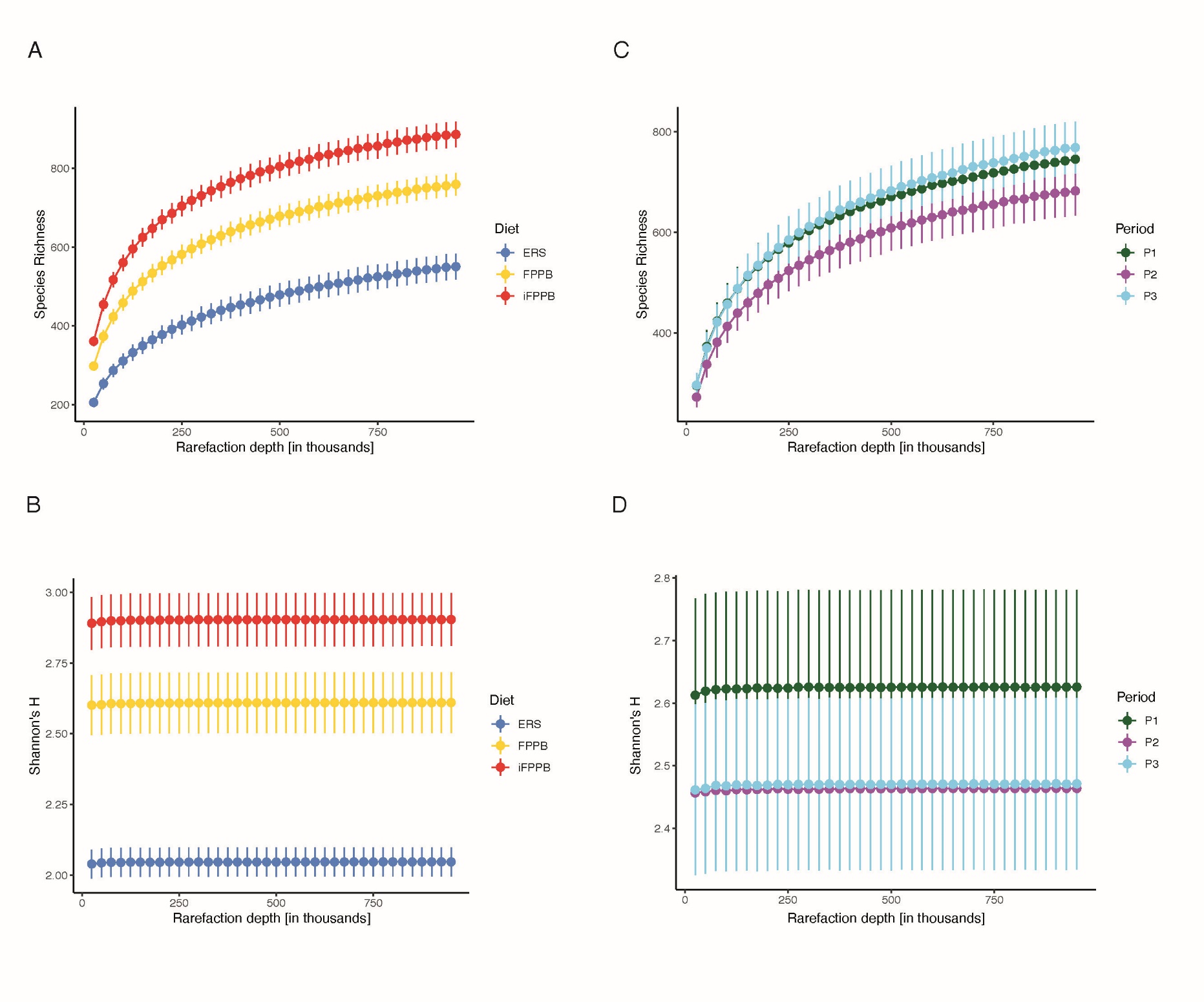


**Supplementary Figure 2.** Rarefaction curves of fecal samples collected from cats fed the experimental diets. The curves show alpha diversity differences between diet groups. **(A, B)** Differences in Species Richness and Shannon’s diversity index (Shannon’s H) between the three diet groups. **(C, D)** No difference in these measures were observed between the periods during which the different diets were administered.


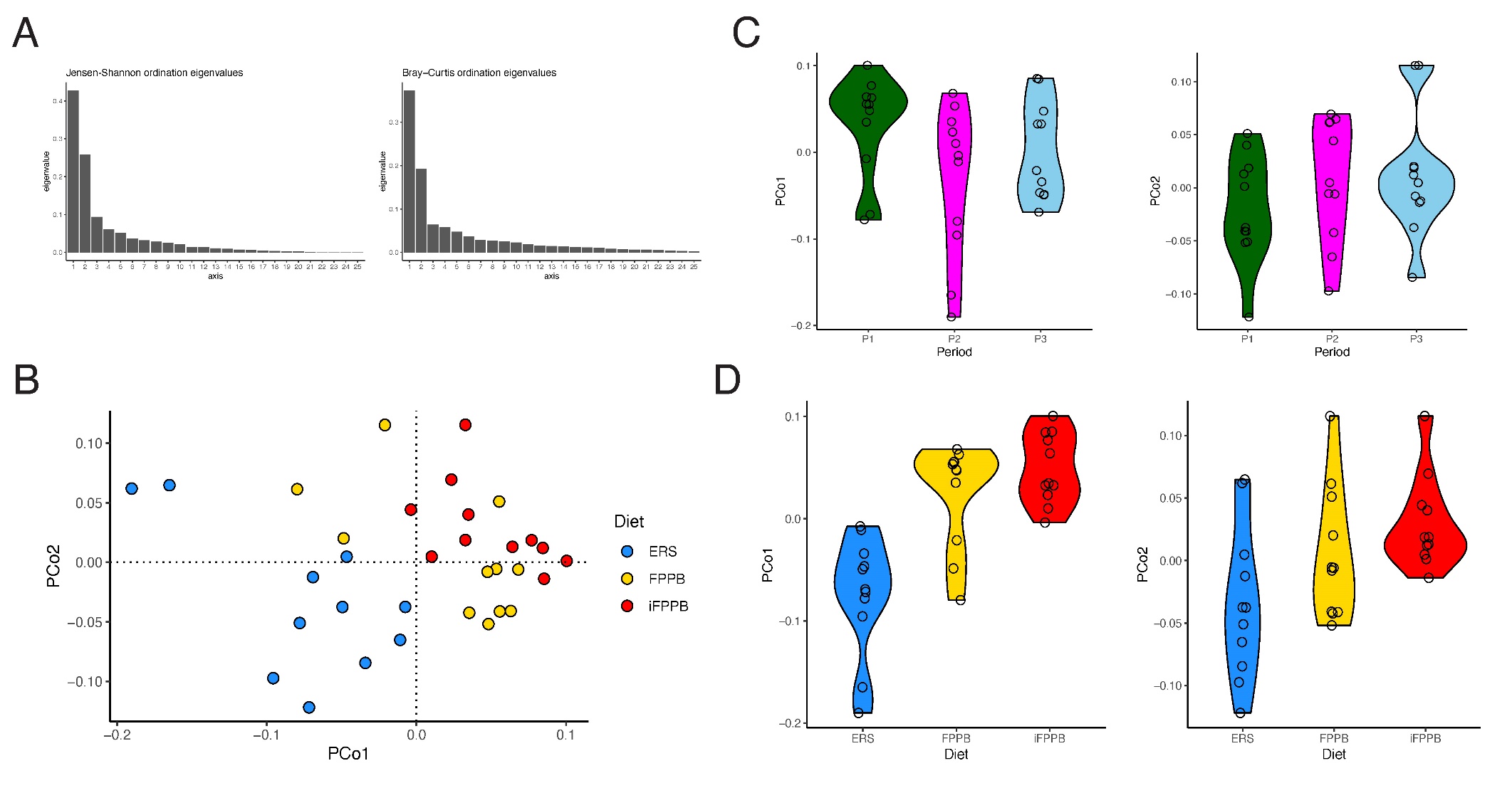


**Supplementary Figure 3.** Fecal microbial composition of cats fed the experimental diets. **(A)** Screeplot showing the eigenvalues obtained from PCoA using Bray-Curtis (left) and Jensen-Shannon distances (right). **(B-D)** Principal Coordinate Analysis (PCoA) of species-level gut microbiomes using Jensen-Shannon distances. PERMANOVA was used to assess the association between diet and period with the gut microbiome composition. Kruskal-Wallis test was used to assess association between diet and the top two axes (PCo1, PCo2) followed by Dunn’s post-hoc test to evaluate the difference between the dietary groups. **(B)** A PCoA plot using Jensen-Shannon distance revealed significant association between diet groups (P=0.0001, PERMANOVA). Period was also significant in this case but with much weaker significance (P=0.034, PERMANOVA). **(C)** No difference in PCo1 or PCo2 between the periods (P=0.12 and 0.41, respectively, Kruskal-Wallis test). **(D)** Diet was associated with both PCo1 and PCo2 (P<0.0001 and 0.03, respectively, Kruskal Wallis test).


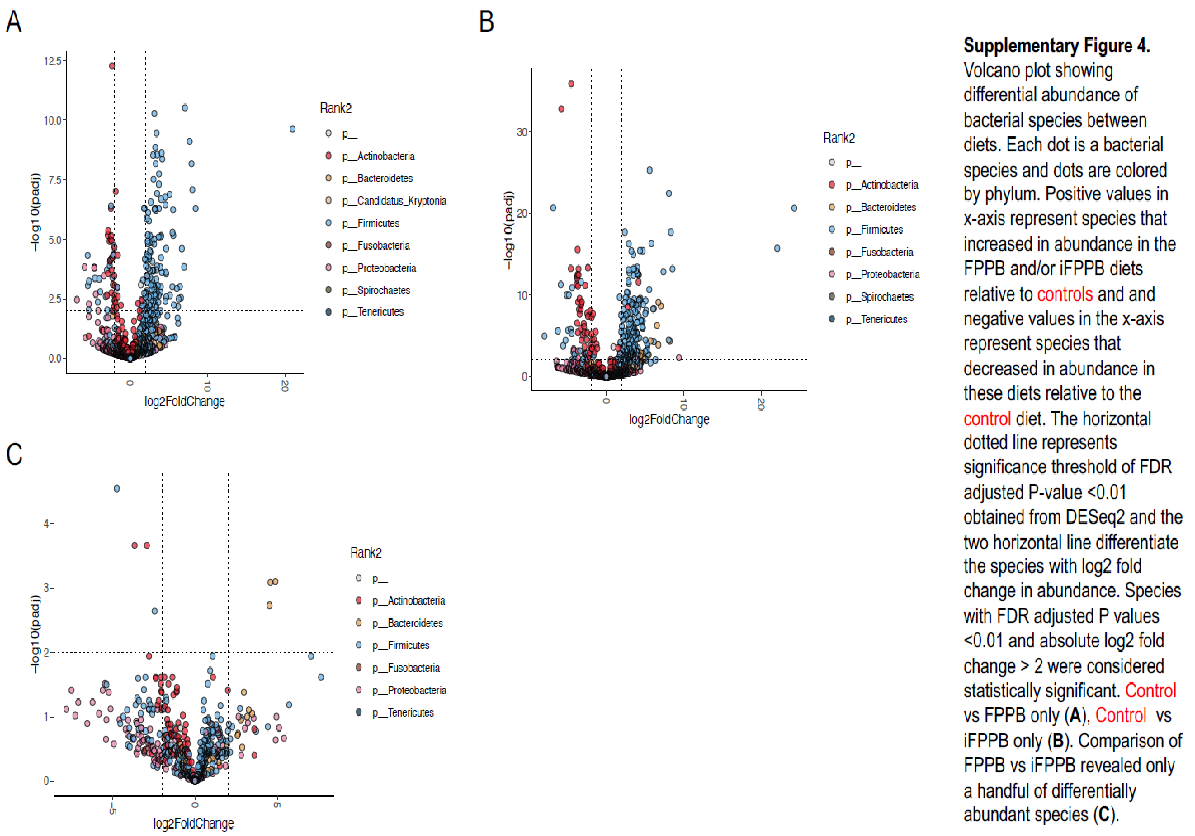


**Supplementary Figure 4.** Volcano plots of fecal microbiota data from cats fed the experimental diets. The plots show differential abundance of bacterial species between diets. Each dot is a bacterial species and dots are colored by phylum. Positive values in x-axis represent species that increased in abundance in the FPPB and/or iFPPB diets relative to ERS and negative values in the x-axis represent species that decreased in abundance in these diets relative to ERS. The horizontal dotted line represents significance threshold of FDR adjusted P-value <0.01 obtained from DESeq2 and the two horizontal lines differentiate the species with log2 fold change in abundance. Species with FDR adjusted P values <0.01 and absolute log2 fold change > 2 were considered statistically significant. ERS vs FPPB only **(A)**, ERS vs iFPPB only **(B)**. Comparison of FPPB vs iFPPB revealed only 7 of differentially abundant species **(C)**.

**Supplementary Figure 5.** Partition Around Medoids clustering analysis of fecal microbiota data from cats fed the experimental diets. The analysis revealed two clusters, with Cluster 1 containing primarily FPPB and iFPPB diet groups and Cluster 2 containing primarily the ERS group.
